# Comparative Analysis of Metagenomic Next-Generation Sequencing, Sanger Sequencing, and Conventional Culture for Detecting Common Pathogens Causing Lower Respiratory Tract Infections in Clinical Samples

**DOI:** 10.3390/microorganisms13030682

**Published:** 2025-03-18

**Authors:** Qiaolian Yi, Ge Zhang, Tong Wang, Jin Li, Wei Kang, Jingjia Zhang, Yali Liu, Yingchun Xu

**Affiliations:** 1 Department of Laboratory Medicine, Peking Union Medical College Hospital, Chinese Academy of Medical Sciences and Peking Union Medical College, Beijing 100730, China; 2 Graduate School, Chinese Academy of Medical Sciences and Peking Union Medical College, Beijing 100730, China

**Keywords:** metagenomic next-generation sequencing, respiratory tract infection, bronchoalveolar lavage fluid, sputum

## Abstract

Metagenomic next-generation sequencing (mNGS) has emerged as a revolutionary tool for infectious disease diagnostics. The necessity of mNGS in real-world clinical practice for common Lower Respiratory Tract Infections (LRTI) needs further evaluation. A total of 184 bronchoalveolar lavage fluid (BALF) samples and 322 sputa associated with LRTI were fully examined. The detection performance was compared between mNGS and standard microbiology culture, using Sanger sequencing as the reference method. 52.05% (165/317) of sputa showed identical results for all three methods. Compared to Sanger sequencing, the same results obtained by mNGS were 88.20% (284/322). In 2.80% (9/322) of cases, Sanger sequencing detected more microorganisms, while mNGS detected more in 9% (29/322) of cases. For BALF, 49.41% (85/172) of cases showed identical results for all three methods. In 91.30% (168/184) of cases, identical results were produced by both mNGS and Sanger sequencing. mNGS detected more species in 7.61% (14/184) of cases, whereas in 2.80% (2/184) instances, the Sanger sequencing detected more microorganisms than mNGS. In the 184 BALF samples, 66 samples were identified as having co-infections by mNGS, Sanger sequencing identified 64 samples, and cultures identified 22 samples. Our study demonstrates that mNGS offers a significant advantage over conventional culture methods in detecting co-infections. For common bacterial pathogens, conventional culture methods are sufficient for detection. However, mNGS provides comprehensive pathogen detection and is particularly useful for identifying rare and difficult-to-culture pathogens.

## 1. Introduction

According to the World Health Organization (WHO), lower respiratory tract infections (LRTI) remained the world’s most deadly communicable disease and claimed 2.6 million lives in 2019 globally [1]. Especially for children younger than 10 years, LRTI ranked as the second leading cause of death [2]. Severe infections have a rapid onset and progression, and the pathogens involved are complex. Pathogens causing LRTI vary from bacteria and fungi to viruses. Identifying the pathogenic microorganisms within a short period is crucial [3].

Traditional methods for microbial identification in clinical practice are divided into culture and non-culture categories [4]. However, culture methods are limited by long operational cycles and high failure rates, and not all pathogens can be cultured. Culture-independent methods with strong timeliness, such as smear microscopy, antibody-antigen immunoassays, and nucleic acid amplification testing (NAAT), can report results on the day of sampling, but prior knowledge of the suspected pathogens is necessary [5]. Metagenomic next-generation sequencing (mNGS) has settled those issues. It enables unbiased sampling and achieves broad identification of known and unexpected pathogens, even discovering new organisms [6].

As a culture-free, whole-organism detecting technique, mNGS has demonstrated applicability as a revolutionary tool in the field of infectious disease diagnostics, especially for unexplained infections [7,8]. Applying mNGS workflows to LRTI has improved the etiological diagnosis of respiratory pathogens [9,10]. However, despite its advantages, mNGS encounters more challenges in identifying the appropriate cases due to the limitation of technical complexity, high costs, sample handling and data analysis, and the ongoing need for extensive clinical validation [11]. The technical complexity of mNGS makes it unsuitable as a frontline detection method [12].

mNGS is supplementary to, rather than a priority over, conventional methods. Previous studies have focused on comparing the detection performance of mNGS with conventional microbial testing (CMT) [13] and demonstrate the diagnostic value of mNGS [14]. The detection of unexpected or difficult-to-detect pathogens by mNGS is also highlighted [15,16,17]. In terms of pathogens detection, there is no doubt that mNGS has a distinct advantage. However, not all microorganisms detected by mNGS are pathogenic with clinical relevance [18]. Additionally, for LRTI caused by common pathogens that can be identified through routine culture or syndromic multiplex polymerase chain reaction (PCR), the necessity for mNGS is not well-established [19].

In this study, we selected 18 typical pathogens of LRTI based on their clinical relevance and prevalence [20,21]. Specifically, we included pathogens that are prone to hospital-acquired infections (such as *Klebsiella pneumoniae*, *Pseudomonas aeruginosa*, *Acinetobacter baumannii*, and *Staphylococcus aureus*), those commonly causing community-acquired infections (such as *Haemophilus influenzae*, *Streptococcus pneumoniae*, *Moraxella catarrhalis*, and *Legionella pneumophila*), pathogens susceptible in immunocompromised populations (such as *Candida albicans*, *Candida tropicalis*, *Candida glabrata*, *Aspergillus fumigatus*, *Pneumocystis jirovecii*, *Cryptococcus neoformans*, *Talaromyces marneffei*, and *Nocardia asteroides*), and others (such as human adenovirus and *Mycoplasma pneumoniae*). The selection of these pathogens aimed to comprehensively cover the common causes of LRTI in various clinical settings and patient populations.

We utilized real clinical samples targeting specific pathogens and compared the detection performance of culture, mNGS, and Sanger sequencing for both sputum and bronchoalveolar lavage fluid (BALF) samples. This way, we aim to provide an overall assessment of which specimens and methods are more time-efficient for individuals with respiratory symptoms, analyzing the necessity of mNGS in real-world clinical practice for common pathogens of LRTI.

## 2. Materials and Methods

### 2.1. Study Population and Specimen

In this prospective, observational, method-comparison clinical study, 184 BALF and 322 sputum samples were collected according to standardized operating procedures in a clinical setting between October 2021 and November 2022. Inclusion was based on the presence of one or more symptoms indicative of a respiratory tract infection: (1) Patients with severe respiratory conditions, such as severe pneumonia, respiratory failure, acute respiratory distress syndrome, and acute exacerbations of chronic obstructive pulmonary disease; (2) Immunocompromised patients with respiratory-related symptoms, including those with congenital immunodeficiencies, secondary immunoglobulin deficiencies (e.g., AIDS patients with HIV infection), individuals undergoing cancer chemotherapy or radiotherapy, organ transplant recipients, or those treated with immunosuppressive agents due to other medical conditions; (3) Special patients with respiratory-related symptoms, characterized by frequent hospitalizations, repeated negative tests, recurrent fevers, or fevers of unknown origin; (4) Critically ill patients with respiratory-related symptoms due to infections, such as bloodstream infections, sepsis, septic shock, and organ failures caused by infections; (5) Critically ill patients with respiratory-related symptoms in settings such as the intensive care unit (ICU), cancer, non-infectious shock/organ failure; (6) Patients with underlying diseases presenting respiratory symptoms, mainly including conditions like cerebral infarction, hypertension, diabetes mellitus, hyperlipidemia, hyperhomocysteinemia, heart disease, coronary heart disease, chronic bronchitis, chronic pneumonia, chronic obstructive pulmonary disease, pulmonary interstitial diseases, chronic cor pulmonale, bronchial asthma, and chronic lung abscesses; (7) Patients with other respiratory symptoms.

Specimen delivered to the microbiology laboratory underwent standard microbiological culture assays. Bacterial culture media used for the isolation and identification of respiratory pathogens include blood agar, chocolate agar, and McConkey agar. Fungal culture media used include CHROMagar Candida and Sabouraud agar. Isolates from positive cultures were identified using MALDI-TOF mass spectrometry (Autof MS1000, Zhengzhou, Henan, China). Nucleic acid extraction was performed on all collected samples, which were then divided into two portions. One portion was used for Sanger sequencing, while the other was utilized for mNGS. Concurrently, a retrospective examination of the clinical culture results for the same samples was conducted.

### 2.2. Process of Sanger Sequencing

Each sample was individually amplified using specific primers for PCR (Supplementary File: Table S1. Primer information for Sanger sequencing). The Sanger sequencing, including the step of PCR band purification, was outsourced to a professional sequencing service provider, Sangon Biotech Co., Ltd. (Shanghai, China). They were responsible for the entire process of Sanger sequencing, including PCR band purification, using their standard protocols. To be specific, the PCR products were loaded into wells of an agarose gel, and electrophoresis was conducted at a constant voltage of 150 V. The electrophoresis was halted when the bromophenol blue indicator dye reached the two-thirds position within the gel. The gel imaging system was used to capture images, and the sample numbers with the targeted bands were recorded. The PCR bands of interest were then excised from the gel and recovered for subsequent sequencing work. The obtained sequencing sequences were aligned using the NCBI BLAST program (https://blast.ncbi.nlm.nih.gov/Blast.cgi, accessed on 11 August 2022).

### 2.3. Process of mNGS

The samples for mNGS were obtained using the Respiratory Pathogen Multiplex Detection Kit (Vision Medicals, Inc., Guangzhou, China). Specifically, DNA nucleic acid was extracted from the samples to be tested; the nucleic acid was fragmented, subjected to end repair and splicing, followed by amplification with primers with tag sequences to complete the library construction. After library quantification, quality control, and cyclization, high-throughput sequencing was performed using a VisionSeq 1000 sequencing platform (Vision Medicals, Inc., Guangzhou, China) gene sequencer. The resulting sequencing data were compared with the pathogen database provided by the nucleic acid analysis software, automated IDseqTM-2 bioinformatic analysis (Vision Medicals, Inc., Guangzhou, China), to analyze the presence of the corresponding pathogens in the samples.

### 2.4. Criteria for Identification and Discrepant Analysis

The threshold criteria for reporting positive results via mNGS were based on the requirements of the product manual. The product manual, combined with the analysis of the receiver operating characteristic (ROC) curve, determined the positive values for each tested pathogen. These values were integrated into the metagenomic analysis software (IDseqTM-2 bioinformatic analysis), which automatically interprets test results. Specifically, the threshold criteria for reporting via mNGS were as follows: for *Mycoplasma pneumoniae*, *Cryptococcus neoformans*, *Talaromyces marneffei*, *Pneumocystis jirovecii*, human adenovirus and *Aspergillus fumigatus*, a threshold of RPM ≥ 0.1 was applied; for other microorganisms, the threshold was RPM ≥ 1. RPM (Reads Per Million) refers to the number of readings of the pathogen detected per million reads in a sample, and serves as a measure of pathogen abundance.

Culture and Sanger sequencing were regarded as reference methods. In the case of discrepancies between culture and Sanger sequencing results, the Sanger sequencing result was considered the final correct identification.

### 2.5. Statistical Analysis

In our study, sensitivity was calculated as the ratio of samples that are positive by both mNGS and culture/Sanger sequencing to the total number of culture/Sanger sequencing-positive samples; specificity was calculated as the ratio of samples that are negative by both mNGS and culture/Sanger sequencing to the total number of culture/Sanger sequencing-negative samples; accuracy was calculated as the ratio of samples with consistent results between mNGS and culture/Sanger sequencing to the total number of samples. Based on true positive (TP), true negative (TN), false positive (FP), and false negative (FN) results for pathogen detection using culture/Sanger sequencing results as the reference, the sensitivity [TP/(TP + FN)], specificity [TN/(TN + FP)], and accuracy [(TP + TN)/(TP + FP + TN + FN)], as well as the corresponding 95% confidence intervals (CI) were calculated using the epitools package in R (version 4.4.3). Concurrently, positive and negative predictive values (PPV, NPV) as well as the positive and negative likelihood ratio (PLR, NLR) were also calculated.

## 3. Results

### 3.1. Patient Characteristics

This study included subjects of both genders and all age groups. A total of 506 specimens were collected, including 184 BALF and 322 sputa. The characteristics of those patients are shown in Table 1. In cases where a clinical diagnosis may fit into multiple categories from 1 to 7, subjects will be classified into only one diagnostic category according to the order of priority from 1 to 7. For BALF, patients with severe respiratory conditions account for most of the clinical types (121/184, 65.76%), followed by patients with underlying diseases presenting respiratory symptoms (21/184, 11.41%). For sputum, patients with severe respiratory conditions account for most of the clinical types (117/322, 36.34%), followed by critically ill patients with respiratory-related symptoms (105/322, 32.61%). Special patients with respiratory-related symptoms were less common for both BALF (2/184, 1.09%) and sputum (2/322, 0.62%) samples.

### 3.2. Test Performance of Culture, Sanger Sequencing, and mNGS

**Figure 1:**
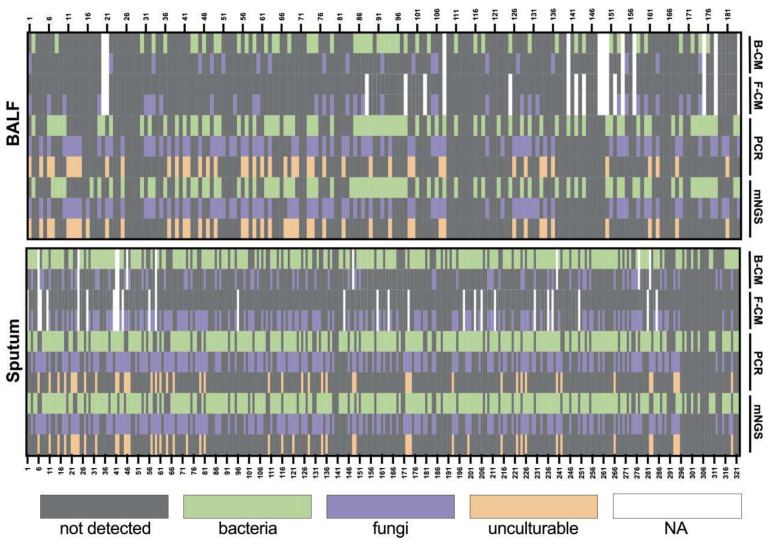
Heatmap depicting the identification of pathogens in BALF and sputum samples by different methods. B-CM, bacterial culture; F-CM, fungal culture.

We evaluated the test performance of the culture, Sanger sequencing, and mNGS separately for 184 BALF and 322 sputum samples (Figure 1). The culture method (CM) includes bacterial cultures (B-CM) and fungal cultures (F-CM). Sanger sequencing results were obtained after PCR. For the sputum samples, 5 samples had no culture results. Out of 317 samples, 165 samples obtained similar results for all three detection methods. Overall agreement rates were 88.20% (284/322) between mNGS and Sanger sequencing, 52.68% (167/317) mNGS and culture, and 57.10% (181/317) between Sanger sequencing and culture. Additionally, Sanger sequencing detected 40 *Pneumocystis jirovecii*, 3 *Legionella pneumophila*, and 3 human adenoviruses. The consistency of NGS and culture and Sanger and culture were increased to 59.94% (190/317) and 65.30% (207/317), respectively, excluding the results of *Pneumocystis jirovecii*, *Legionella pneumophila*, and human adenovirus.

For the BALF samples, culture results were unavailable for 12 samples, and 85 out of 172 samples obtained similar results among tested methods. The agreement rates were 91.30% (168/184), 54.65% (94/172), and 56.98% (98/172), respectively, between mNGS and Sanger sequencing, mNGS and culture, and Sanger sequencing and culture. *Pneumocystis jirovecii* and *Legionella pneumophila* detected by Sanger sequencing appeared among 40 samples and 3 samples, respectively. No human adenovirus was detected for the 184 BALF samples. Specifically, *Pneocumystis jirovecii*, *Legionella pneumophila*, and human adenovirus are known to be non-culturable or difficult to culture using universal microbiological culture media. Thus, the consistency of NGS and culture and Sanger and culture were increased to 68.60% (118/172) and 73.84% (127/172), respectively, excluding the results of *Pneumocystis jirovecii* and *Legionella pneumophila*.

### 3.3. Co-Infection Detected in BALF

Among the 184 BALF samples, 66 samples were identified as having co-infections were identified in 66 samples by mNGS, 64 samples by Sanger sequencing, and 22 samples by culture. The most frequently isolated pathogens in co-infections were as follows: *Acinetobacter baumannii* (27/64 by Sanger, 30/66 by mNGS), *Pneumocystis jirovecii* (24/64, 25/66), *Candida albicans* (23/64, 17/66), *Pseudomonas aeruginosa* (23/64, 26/66), *Klebsiella pneumoniae* (16/64, 17/66), *Aspergillus fumigatus* (13/64, 13/66), and *Staphylococcus aureus* (9/64, 7/66). Regarding the most common co-infection combinations, the highest was *Candida albicans* with *Acinetobacter baumannii* (11/64 by Sanger, 9/66 by mNGS), followed by *Pneumocystis jirovecii* with *Aspergillus fumigatus* (7/64, 7/66), and then *Pneumocystis jirovecii* with *Acinetobacter baumannii* (6/64, 7/66). Co-infections involving both viral and bacterial infections were not detected in this study. However, it should be noted that this study only tested for 18 pathogens, with human adenovirus being the only virus included.

### 3.4. Comparison of Culture, Sanger Sequencing, and mNGS in Certain Microorganisms

We selected 18 of the most typical pathogens associated with clinical respiratory infections to analyze and compare their detection using mNGS, Sanger sequencing, and conventional culture methods. Table 2 and Table 3 present the statistical analysis of the detection of specific pathogens in the samples by mNGS compared to culture and mNGS compared to Sanger sequencing, including the number of detections, sensitivity, specificity, overall agreement rate, and Kappa values. In our study, sensitivity was calculated as the ratio of samples that tested positive by both mNGS and culture/Sanger sequencing to the total number of culture/Sanger sequencing-positive samples. Specificity was calculated as the ratio of samples that are negative by both mNGS and culture/Sanger sequencing to the total number of culture/Sanger sequencing-negative samples. Accuracy was calculated as the ratio of samples with consistent results between mNGS and culture/Sanger sequencing to the total number of samples. Additionally, positive and negative predictive values, as well as the likelihood ratio, were also calculated.

Compared with the culture method (Table 2), the specificity of *Acinetobacter baumannii* in BALF samples was 88.28%, while the specificity of the other 13 cultivable pathogens exceeded 93%. The overall agreement rate for all pathogens was above 90%. Notably, no positive samples were detected by culture for *Talaromyces marneffei*, *Haemophilus influenzae*, *Streptococcus pneumoniae*, *Moraxella catarrhalis*, and *Nocardia asteroides*. For the remaining 9 pathogens, the sensitivity was above 95%. In sputum samples, the specificity for all pathogens was above 90%, and the overall agreement rate was above 91%; among them, no positive samples were detected by culture for *Talaromyces marneffei* and *Nocardia asteroides*. For the other 12 pathogens, the sensitivity was greater than 96%.

Compared with Sanger sequencing, for all 18 pathogens, the clinical specificity was above 96% in BALF samples (Table 3), and the overall agreement rate was above 97%. Among them, no positive samples were detected for *Talaromyces marneffei*, *Nocardia asteroides*, *Haemophilus influenzae*, *Moraxella catarrhalis*, *Mycoplasma pneumoniae*, and human adenovirus. For the other 12 pathogens, the sensitivity was above 96%. The Kappa value was above 0.75, indicating good consistency between mNGS and Sanger sequencing. In sputum samples, the clinical specificity was above 97%, and the overall agreement rate was above 97% for all 18 pathogens. Among them, no positive samples were detected for *Talaromyces marneffei*, *Nocardia asteroides*, and *Mycoplasma pneumoniae*. Human adenovirus was detected in three cases by Sanger sequencing, while mNGS detected only one case, which was not statistically significant due to the small sample size. For the other 14 pathogens, the clinical sensitivity was above 96%; the Kappa value was above 0.75, indicating good consistency between mNGS and Sanger sequencing.

## 4. Discussion

The purpose of this study was to analyze whether there is a necessity to use mNGS for detecting typical clinical respiratory infection pathogens, and whether traditional culture or PCR methods are sufficient for clinical applications. We chose the 18 most typical respiratory pathogens for analysis and compared the detection rates of mNGS, Sanger sequencing, and conventional culture in both sputum and BALF samples.

Based on the guidelines from IDSA and ASM, the optimum specimens for laboratory diagnosis of LRTI vary depending on the type of respiratory infection [22]. Sputum specimens are commonly used for the diagnosis of Community-Acquired Pneumonia (CAP), while both sputum and BALF samples are recommended for the diagnosis of Hospital-Acquired Pneumonia (HAP) and Ventilator-Associated Pneumonia (VAP). Bacterial cultures from BALF can be used to assess patients with artificial airways for LRTI [23]. Among the approved diagnostic products, there are also those that directly use sputum and BALF as specimens for respiratory pathogen detection, such as the BioFire^®^ FilmArray Pneumonia (PN) Panel [21]. In studies utilizing mNGS for pathogen detection and diagnosis in LRTI, BALF alone accounted for 87.80% specimens, highlighting its significant role as a diagnostic sample type [13]. Reviews on the influence of different sampling methods on the detection efficiency of mNGS showed that BALF mNGS was better for detection of bacteria and fungi than blood mNGS in general [14].

In this study, we demonstrated the diagnostic efficiency of mNGS to detect pathogens causing LRTI. (1) Although for a single patient, mNGS can provide a more comprehensive coverage of potential pathogens than conventional microbiologic test, the majority of pathogens were identified with conventional culture method and PCR (Sanger sequencing). (2) Between the two different specimens, sputum seemed to have superior performance over BALF for common pathogen detection. (3) Sanger sequencing seemed to be generally sufficient to cover common respiratory pathogens, making the use of mNGS unnecessary in many cases.

As seen in this study, samples with completely consistent Sanger sequencing and culture results, as well as mNGS results, account for only half of all samples (52.05% in sputum and 49.42% in BALF). This confirms the limitations of traditional culture methods in detecting pathogens. mNGS testing identifies a broader range of pathogens than conventional methods [8], and performs well in identifying rare, novel, difficult-to-detect and co-infected pathogens directly from clinical samples [6]. In addition, assessment of mNGS for unbiased microbe detection among multi-laboratory has also been evaluated [24]. However, limitations associated with such a powerful technology are also evident. The cost of mNGS testing varies depending on the specific platform and the volume of samples. According to a study published in 2021, the cost of mNGS testing can range from $1000 to $2500 per test [25]. The cost of mNGS for cerebrospinal fluid samples can be up to $3000 per sample [26]. These costs are significantly higher than those of conventional microbiological tests. Otherwise, the overall operational process of mNGS is complex, the equipment is expensive, and requires highly skilled operators, as well as strict operating environments. The critical considerations for practical implementation of mNGS assays (e.g., pre-analysis phase, analysis phase and post-analysis phase) and quality management in a clinical environment (e.g., test validation, quality control procedures and proficiency testing) can pose another barrier to the widespread adoption of mNGS in clinical laboratories [11] Therefore, mNGS may not be a feasible or affordable option for all laboratories.

Besides, the interpretation of mNGS results lacks standardization, making its results more cautious to assess. From our results, it can be seen that for common pathogens, the consistency between mNGS and Sanger sequencing in sputum samples was 88.2%, further increasing to 91.3% in BALF samples (Supplementary File: Tables S2 and S3. Microorganisms detected by mNGS and Sanger sequencing). The inconsistencies are mainly attributed to the detection principles. In samples with a low pathogen load, Sanger sequencing may fail to amplify the target fragment, leading to missed detections. Similarly, in mNGS, a high proportion of human cells may hinder the detection of low-abundance pathogens in weakly positive samples [27].

Syndromic multiplex polymerase chain reaction (PCR) panels that enable the simultaneous detection of multiple pathogens in a single test have been a significant advancement in diagnostic medicine [28,29]. While mNGS provides a comprehensive and unbiased view of the microbial landscape, multiplex PCR is valued for its specificity and rapid results. For detecting common pathogens causing LRTI, multiplex PCR can meet the clinical demand, potentially offering a more accessible and quicker alternative to mNGS for the diagnosis of common pathogens.

Thus, the results of our study suggest that: For HAP and VAP patients, we do not recommend using mNGS as a first-line choice. This is because conventional microbiological methods are sufficient for detecting common bacterial pathogens in these cases. The high cost and technical complexity of mNGS make it less suitable as a routine diagnostic tool for these patients. However, in cases where conventional microbiological methods fail, mNGS can be a valuable tool. This includes patients with severe infections who need a rapid and accurate diagnosis, patients with immunocompromised conditions where conventional methods may not be sensitive enough, and patients with clustered respiratory infectious diseases where pathogen tracing is necessary. Our study shows that mNGS, including multiplex PCR methods, has a better diagnostic efficiency in co-infections. While mNGS offers significant advantages in terms of diagnostic accuracy and speed, its high cost may limit its widespread use in routine clinical practice. Therefore, it is crucial to develop clear criteria on when and how to use mNGS in hospital settings to maximize its value while minimizing unnecessary expenses.

Our study has certain limitations. Firstly, due to issues in the clinical diagnostic process, the specimens tested in this study were residual specimens from clinical testing. Some of these samples underwent pathogen microbial culture, and culture reports were issued. Fungi and bacteria were cultured separately. Therefore, the number of samples analyzed for different pathogens varies. Additionally, adenovirus, *Mycoplasma pneumoniae*, *Legionella pneumophila* are difficult to culture, and *Pneumocystis jirovecii* cannot be cultured. No culture results for these four types of pathogens, hence no comparative analysis between the mNGS and culture results was conducted for these pathogens. Without interfering with clinical diagnosis and treatment, this led to a bias in the patient grouping for the performance of the two types of specimens tested (sputum and BALF), as they were not from the same patient. Secondly, the specimens used in this study were part of a cross-sectional study, where patients may have already received antifungal, antibacterial, or antiviral treatment, which could also affect the sensitivity of the detection. Lastly, further matched cohort studies are needed to determine which testing method is more suitable for specific patient populations to address different diagnostic and treatment scenarios. However, population classification analysis was not conducted in this study.

From our results, it can be seen that there is a high correlation between the number of bacteria cultured and the reads per million (RPM) detected by NGS. Therefore, for common pathogens, the high abundance that can be detected by NGS can also be achieved by culture methods. Our results once again confirm that NGS is suitable for situations where pathogens are difficult or impossible to culture and for the detection of rare pathogens. It is suitable as a supplement to conventional detection methods, rather than a replacement.

## 5. Conclusions

This study provides valuable insights into the diagnostic capabilities of mNGS, Sanger sequencing, and conventional culture methods for lower respiratory tract infections. While mNGS offers comprehensive pathogen detection and is particularly useful for identifying rare and difficult-to-culture pathogens, its high cost and technical complexity make it less suitable as a frontline diagnostic tool. Sanger sequencing and conventional culture methods remain effective for detecting common respiratory pathogens, suggesting that mNGS may not be necessary in many routine clinical scenarios. The study highlights the importance of selecting appropriate diagnostic methods based on the specific clinical context and pathogen characteristics. Further research is needed to refine the application of mNGS and to establish standardized interpretation guidelines, ensuring its optimal use in clinical practice.

## Figures and Tables

**Table 1:** Characteristics of the patients with symptoms indicative of respiratory tract infection.

Issues	BALF (*n* = 184)	Sputum (*n* = 322)
Sex, male, n (%)	116 (63.04%)	207 (64.29%)
Age at onset, y, median (range)	57.43 ± 16.703, 59 (13–90)	60.61 ± 16.517, 64 (10–100)
Clinical classification		
Patients with severe respiratory conditions	121 (65.76%)	117 (36.34%)
Immunocompromised patients with respiratory-related symptoms	12 (6.52%)	18 (5.59%)
Special patients with respiratory-related symptoms	2 (1.09%)	2 (0.62%)
Critically ill patients with respiratory-related symptoms due to infections	6 (3.26%)	23 (7.14%)
Critically ill patients with respiratory-related symptoms in settings	6 (3.26%)	105 (32.61%)
Patients with underlying diseases presenting respiratory symptoms	21 (11.41%)	34 (10.56%)
Patients with other respiratory symptoms	16 (8.70%)	23 (7.14%)

**Table 2:** Statistical results of mNGS compared with culture results.

Organism	TP	FP	FN	TN	Sensitivity [95% CI]	Specificity [95% CI]	PPV [95% CI]	NPV [95% CI]	PLR	NLR	Accuracy [95% CI]
BALF											
*Aspergillus fumigatus*	8	10	0	147	1.00 [0.6306, 1]	0.94 [0.886, 0.969]	0.44 [0.2153, 0.6924]	1.00 [0.9752, 1]	15.70	0.00	0.94 [0.8914, 0.9706]
*Talaromyces marneffei*	0	0	0	165	0.00 [0, 0]	1.00 [0.9779, 1]	0.00 [0, 0]	1.00 [0.9779, 1]	NA	0.00	1.00 [0.9779, 1]
*Cryptococcus neoformans*	1	0	0	164	1.00 [0.025, 1]	1.00 [0.9778, 1]	1.00 [0.025, 1]	1.00 [0.9778, 1]	Inf	0.00	1.00 [0.9779, 1]
*Candida albicans*	23	4	1	137	0.96 [0.7888, 0.9989]	0.97 [0.929, 0.9922]	0.85 [0.6627, 0.9581]	0.99 [0.9603, 0.9998]	33.78	0.04	0.97 [0.9307, 0.9901]
*Candida tropicalis*	4	1	0	160	1.00 [0.3976, 1]	0.99 [0.9659, 0.9998]	0.80 [0.2836, 0.9949]	1.00 [0.9772, 1]	161.00	0.00	0.99 [0.9667, 0.9998]
*Candida glabrata*	5	1	0	159	1.00 [0.4782, 1]	0.99 [0.9657, 0.9998]	0.83 [0.3588, 0.9958]	1.00 [0.9771, 1]	160.00	0.00	0.99 [0.9667, 0.9998]
*Haemophilus influenzae*	0	0	0	172	0.00 [0, 0]	1.00 [0.9788, 1]	0.00 [0, 0]	1.00 [0.9788, 1]	NA	0.00	1.00 [0.9788, 1]
*Streptococcus pneumoniae*	0	1	0	171	0.00 [0, 0]	0.99 [0.968, 0.9999]	0.00 [0, 0.975]	1.00 [0.9787, 1]	0.00	0.00	0.99 [0.968, 0.9999]
*Moraxella catarrhalis*	0	0	0	172	0.00 [0, 0]	1.00 [0.9788, 1]	0.00 [0, 0]	1.00 [0.9788, 1]	NA	0.00	1.00 [0.9788, 1]
*Nocardia asteroids*	0	0	0	172	0.00 [0, 0]	1.00 [0.9788, 1]	0.00 [0, 0]	1.00 [0.9788, 1]	NA	0.00	1.00 [0.9788, 1]
*Klebsiella pneumoniae*	10	10	0	152	1.00 [0.6915, 1]	0.94 [0.8894, 0.97]	0.50 [0.272, 0.728]	1.00 [0.976, 1]	16.20	0.00	0.94 [0.8957, 0.9718]
*Acinetobacter baumannii*	27	17	0	128	1.00 [0.8723, 1]	0.88 [0.8189, 0.9302]	0.61 [0.455, 0.7564]	1.00 [0.9716, 1]	8.53	0.00	0.90 [0.8465, 0.9414]
*Pseudomonas aeruginosa*	16	10	0	146	1.00 [0.7941, 1]	0.94 [0.8853, 0.9688]	0.62 [0.4057, 0.7977]	1.00 [0.9751, 1]	15.60	0.00	0.94 [0.8957, 0.9718]
*Staphylococcus aureus*	5	8	0	159	1.00 [0.4782, 1]	0.95 [0.9078, 0.9791]	0.38 [0.1386, 0.6842]	1.00 [0.9771, 1]	20.88	0.00	0.95 [0.9104, 0.9797]
Sputum											
*Aspergillus fumigatus*	29	9	1	256	0.97 [0.8278, 0.9992]	0.97 [0.9365, 0.9844]	0.76 [0.5976, 0.8856]	1.00 [0.9785, 0.9999]	28.46	0.03	0.97 [0.9385, 0.9836]
*Talaromyces marneffei*	0	0	0	295	0.00 [0, 0]	1.00 [0.9876, 1]	0.00 [0, 0]	1.00 [0.9876, 1]	NA	0.00	1.00 [0.9876, 1]
*Cryptococcus neoformans*	1	4	0	290	1.00 [0.025, 1]	0.99 [0.9655, 0.9963]	0.20 [0.0051, 0.7164]	1.00 [0.9874, 1]	73.50	0.00	0.99 [0.9656, 0.9963]
*Candida albicans*	85	15	3	196	0.97 [0.9036, 0.9929]	0.93 [0.8855, 0.9597]	0.85 [0.7647, 0.9135]	0.98 [0.9566, 0.9969]	13.59	0.04	0.94 [0.9065, 0.9639]
*Candida tropicalis*	18	8	0	269	1.00 [0.8147, 1]	0.97 [0.9439, 0.9875]	0.69 [0.4821, 0.8567]	1.00 [0.9864, 1]	34.63	0.00	0.97 [0.9473, 0.9882]
*Candida glabrata*	27	4	0	265	1.00 [0.8723, 1]	0.99 [0.9624, 0.9959]	0.87 [0.7017, 0.9637]	1.00 [0.9862, 1]	67.25	0.00	0.99 [0.9658, 0.9963]
*Haemophilus influenzae*	19	13	0	280	1.00 [0.8235, 1]	0.96 [0.9253, 0.9762]	0.59 [0.4064, 0.763]	1.00 [0.9869, 1]	22.54	0.00	0.96 [0.9298, 0.9776]
*Streptococcus pneumoniae*	9	12	0	291	1.00 [0.6637, 1]	0.96 [0.9318, 0.9794]	0.43 [0.2182, 0.6598]	1.00 [0.9874, 1]	25.25	0.00	0.96 [0.9338, 0.98]
*Moraxella catarrhalis*	5	8	0	299	1.00 [0.4782, 1]	0.97 [0.9493, 0.9887]	0.38 [0.1386, 0.6842]	1.00 [0.9877, 1]	38.38	0.00	0.97 [0.9501, 0.9889]
*Nocardia asteroids*	0	0	0	312	0.00 [0, 0]	1.00 [0.9882, 1]	0.00 [0, 0]	1.00 [0.9882, 1]	NA	0.00	1.00 [0.9882, 1]
*Klebsiella pneumoniae*	46	16	1	250	0.98 [0.8871, 0.9995]	0.94 [0.9042, 0.9652]	0.74 [0.615, 0.8447]	1.00 [0.978, 0.9999]	16.27	0.02	0.95 [0.9145, 0.968]
*Acinetobacter baumannii*	50	19	1	242	0.98 [0.8955, 0.9995]	0.93 [0.8887, 0.9556]	0.72 [0.6038, 0.8254]	1.00 [0.9773, 0.9999]	13.47	0.02	0.94 [0.9027, 0.9604]
*Pseudomonas aeruginosa*	46	25	0	241	1.00 [0.9229, 1]	0.91 [0.8644, 0.9383]	0.65 [0.5254, 0.7576]	1.00 [0.9848, 1]	10.64	0.00	0.92 [0.884, 0.9475]
*Staphylococcus aureus*	40	27	0	245	1.00 [0.9119, 1]	0.90 [0.8589, 0.9336]	0.60 [0.47, 0.7151]	1.00 [0.9851, 1]	10.07	0.00	0.91 [0.8766, 0.9422]

**Table 3:** Statistical results of mNGS compared with Sanger sequencing results.

Organism	TP	FP	FN	TN	Sensitivity [95% CI]	Specificity [95% CI]	PPV [95% CI]	NPV [95% CI]	PLR	NLR	Accuracy [95% CI]
BALF											
*Aspergillus fumigatus*	17	1	0	166	1.00 [0.8049, 1]	0.99 [0.9671, 0.9998]	0.94 [0.7271, 0.9986]	1.00 [0.978, 1]	167.00	0.00	0.99 [0.9701, 0.9999]
*Pneumocystis jirovecii*	40	2	0	142	1.00 [0.9119, 1]	0.99 [0.9507, 0.9983]	0.95 [0.8384, 0.9942]	1.00 [0.9744, 1]	72.00	0.00	0.99 [0.9613, 0.9987]
*Talaromyces marneffei*	0	0	0	184	0.00 [0, 0]	1.00 [0.9802, 1]	0.00 [0, 0]	1.00 [0.9802, 1]	NA	0.00	1.00 [0.9802, 1]
*Cryptococcus neoformans*	1	0	0	183	1.00 [0.025, 1]	1.00 [0.98, 1]	1.00 [0.025, 1]	1.00 [0.98, 1]	Inf	0.00	1.00 [0.9802, 1]
*Nocardia asteroids*	0	0	0	184	0.00 [0, 0]	1.00 [0.9802, 1]	0.00 [0, 0]	1.00 [0.9802, 1]	NA	0.00	1.00 [0.9802, 1]
*Candida albicans*	29	2	1	152	0.97 [0.8278, 0.9992]	0.99 [0.9539, 0.9984]	0.94 [0.7858, 0.9921]	0.99 [0.9641, 0.9998]	74.43	0.03	0.98 [0.9531, 0.9966]
*Candida tropicalis*	5	1	0	178	1.00 [0.4782, 1]	0.99 [0.9693, 0.9999]	0.83 [0.3588, 0.9958]	1.00 [0.9795, 1]	179.00	0.00	0.99 [0.9701, 0.9999]
*Candida glabrata*	6	0	0	178	1.00 [0.5407, 1]	1.00 [0.9795, 1]	1.00 [0.5407, 1]	1.00 [0.9795, 1]	Inf	0.00	1.00 [0.9802, 1]
*Haemophilus influenzae*	0	0	0	184	0.00 [0, 0]	1.00 [0.9802, 1]	0.00 [0, 0]	1.00 [0.9802, 1]	NA	0.00	1.00 [0.9802, 1]
*Streptococcus pneumoniae*	1	0	0	183	1.00 [0.025, 1]	1.00 [0.98, 1]	1.00 [0.025, 1]	1.00 [0.98, 1]	Inf	0.00	1.00 [0.9802, 1]
*Moraxella catarrhalis*	0	0	0	184	0.00 [0, 0]	1.00 [0.9802, 1]	0.00 [0, 0]	1.00 [0.9802, 1]	NA	0.00	1.00 [0.9802, 1]
*Legionella pneumophila*	5	1	0	178	1.00 [0.4782, 1]	0.99 [0.9693, 0.9999]	0.83 [0.3588, 0.9958]	1.00 [0.9795, 1]	179.00	0.00	0.99 [0.9701, 0.9999]
*Klebsiella pneumoniae*	20	1	0	163	1.00 [0.8316, 1]	0.99 [0.9665, 0.9998]	0.95 [0.7618, 0.9988]	1.00 [0.9776, 1]	164.00	0.00	0.99 [0.9701, 0.9999]
*Acinetobacter baumannii*	44	5	0	135	1.00 [0.9196, 1]	0.96 [0.9186, 0.9883]	0.90 [0.7777, 0.966]	1.00 [0.973, 1]	28.00	0.00	0.97 [0.9377, 0.9911]
*Pseudomonas aeruginosa*	25	2	1	156	0.96 [0.8036, 0.999]	0.99 [0.955, 0.9985]	0.93 [0.7571, 0.9909]	0.99 [0.965, 0.9998]	75.96	0.04	0.98 [0.9531, 0.9966]
*Staphylococcus aureus*	12	1	0	171	1.00 [0.7354, 1]	0.99 [0.968, 0.9999]	0.92 [0.6397, 0.9981]	1.00 [0.9787, 1]	172.00	0.00	0.99 [0.9701, 0.9999]
*Mycoplasma pneumoniae*	0	0	0	184	0.00 [0, 0]	1.00 [0.9802, 1]	0.00 [0, 0]	1.00 [0.9802, 1]	NA	0.00	1.00 [0.9802, 1]
human adenoviruses	0	0	0	184	0.00 [0, 0]	1.00 [0.9802, 1]	0.00 [0, 0]	1.00 [0.9802, 1]	NA	0.00	1.00 [0.9802, 1]
Sputum											
*Aspergillus fumigatus*	38	3	1	280	0.97 [0.8652, 0.9994]	0.99 [0.9693, 0.9978]	0.93 [0.8008, 0.9846]	1.00 [0.9803, 0.9999]	91.91	0.03	0.99 [0.9685, 0.9966]
*Pneumocystis jirovecii*	40	3	0	279	1.00 [0.9119, 1]	0.99 [0.9692, 0.9978]	0.93 [0.8094, 0.9854]	1.00 [0.9869, 1]	94.00	0.00	0.99 [0.973, 0.9981]
*Talaromyces marneffei*	0	0	0	322	0.00 [0, 0]	1.00 [0.9886, 1]	0.00 [0, 0]	1.00 [0.9886, 1]	NA	0.00	1.00 [0.9886, 1]
*Cryptococcus neoformans*	5	0	0	317	1.00 [0.4782, 1]	1.00 [0.9884, 1]	1.00 [0.4782, 1]	1.00 [0.9884, 1]	Inf	0.00	1.00 [0.9886, 1]
*Nocardia asteroids*	0	0	0	322	0.00 [0, 0]	1.00 [0.9886, 1]	0.00 [0, 0]	1.00 [0.9886, 1]	NA	0.00	1.00 [0.9886, 1]
*Candida albicans*	99	2	2	219	0.98 [0.9303, 0.9976]	0.99 [0.9677, 0.9989]	0.98 [0.9303, 0.9976]	0.99 [0.9677, 0.9989]	108.31	0.02	0.99 [0.9685, 0.9966]
*Candida tropicalis*	26	1	0	295	1.00 [0.8677, 1]	1.00 [0.9813, 0.9999]	0.96 [0.8103, 0.9991]	1.00 [0.9876, 1]	296.00	0.00	1.00 [0.9828, 0.9999]
*Candida glabrata*	31	0	0	291	1.00 [0.8878, 1]	1.00 [0.9874, 1]	1.00 [0.8878, 1]	1.00 [0.9874, 1]	Inf	0.00	1.00 [0.9886, 1]
*Haemophilus influenzae*	30	2	0	290	1.00 [0.8843, 1]	0.99 [0.9755, 0.9992]	0.94 [0.7919, 0.9923]	1.00 [0.9874, 1]	146.00	0.00	0.99 [0.9777, 0.9992]
*Streptococcus pneumoniae*	21	2	0	299	1.00 [0.8389, 1]	0.99 [0.9762, 0.9992]	0.91 [0.7196, 0.9893]	1.00 [0.9877, 1]	150.50	0.00	0.99 [0.9777, 0.9992]
*Moraxella catarrhalis*	13	1	0	308	1.00 [0.7529, 1]	1.00 [0.9821, 0.9999]	0.93 [0.6613, 0.9982]	1.00 [0.9881, 1]	309.00	0.00	1.00 [0.9828, 0.9999]
*Legionella pneumophila*	3	0	0	319	1.00 [0.2924, 1]	1.00 [0.9885, 1]	1.00 [0.2924, 1]	1.00 [0.9885, 1]	Inf	0.00	1.00 [0.9886, 1]
*Klebsiella pneumoniae*	58	4	2	258	0.97 [0.8847, 0.9959]	0.98 [0.9614, 0.9958]	0.94 [0.843, 0.9821]	0.99 [0.9725, 0.9991]	63.32	0.03	0.98 [0.9599, 0.9931]
*Acinetobacter baumannii*	66	5	2	249	0.97 [0.8978, 0.9964]	0.98 [0.9547, 0.9936]	0.93 [0.8433, 0.9767]	0.99 [0.9715, 0.999]	49.31	0.03	0.98 [0.9557, 0.9912]
*Pseudomonas aeruginosa*	68	3	1	250	0.99 [0.9219, 0.9996]	0.99 [0.9657, 0.9975]	0.96 [0.8814, 0.9912]	1.00 [0.978, 0.9999]	83.11	0.01	0.99 [0.9685, 0.9966]
*Staphylococcus aureus*	61	7	0	254	1.00 [0.9413, 1]	0.97 [0.9455, 0.9892]	0.90 [0.7993, 0.9576]	1.00 [0.9856, 1]	37.29	0.00	0.98 [0.9557, 0.9912]
*Mycoplasma pneumoniae*	0	0	0	322	0.00 [0, 0]	1.00 [0.9886, 1]	0.00 [0, 0]	1.00 [0.9886, 1]	NA	0.00	1.00 [0.9886, 1]
human adenoviruses	2	1	1	318	0.67 [0.0943, 0.9916]	1.00 [0.9827, 0.9999]	0.67 [0.0943, 0.9916]	1.00 [0.9827, 0.9999]	212.67	0.33	0.99 [0.9777, 0.9992]

## Data Availability

Data available on request due to ethical restrictions.

## Supplementary Materials

The following supporting information can be downloaded at: https://www.mdpi.com/article/10.3390/microorganisms13030682/s1, Table S1: Primer information for Sanger sequencing; Table S2: Microorganisms detected by mNGS and Sanger sequencing in BALF; Table S3: Microorganisms detected by mNGS and Sanger sequencing in sputum.

## Abbreviations

The following abbreviations are used in this manuscript:

mNGS	Metagenomic next-generation sequencing
LRTI	Lower Respiratory Tract Infections
BALF	bronchoalveolar lavage fluid
PCR	polymerase chain reaction
