# Supplementary material for: Comparative Analysis of Metagenomic Next-Generation Sequencing, Sanger Sequencing, and Conventional Culture for Detecting Common Pathogens Causing Lower Respiratory Tract Infections in Clinical Samples
Source: Microorganisms. 2025 Mar 18;13(3):682. doi: 10.3390/microorganisms13030682 (PMC11944894; doi:10.3390/microorganisms13030682)
Supplement: Supplementary file 1 [file microorganisms-13-00682-s001.zip › microorganisms-3493019-supplementary.pdf]

Table S1: Primer information for Sanger sequencing

Microorganism	Primer	Oligonucleotide sequences (5'-3')	bp	GC%	Tm/℃	Molecular Weight	Target gene
Acinetobacter baumannii	aba_F1	TACATTAATTACAGCACTTGG	21	33.33%	46.73	6404.26	gyrB
Acinetobacter baumannii	aba_R1	CAGCACTAATATGTAAAGCAA	21	33.33%	47.03	6431.29	gyrB
Acinetobacter baumannii	aba_F2	GTTCGCACATCCGTACATTG	20	50.00%	54.22	6068.01	gyrB
Haemophilus influenzae	hin_F2	AAGGGTTATTGGGTAAACTATA	22	31.82%	47.14	6837.54	protein D
Haemophilus influenzae	hin_R1	AAGTTCTTTTACCAACGGAGT	21	38.10%	51.01	6420.25	protein D
Klebsiella pneumonia	kpn_F1	TTCAAGGAGGAAACATGCGTA	21	42.86%	53.70	6503.32	hypothetical protein
Klebsiella pneumonia	kpn_R1	CTGGACTTATCGACCGCATC	20	55.00%	55.68	6053.00	hypothetical protein
Klebsiella pneumonia	kpn_F2	AGCCTATCTGAAACAACATCTCA	23	39.13%	53.10	6960.63	hypothetical protein
Legionella pneumophila	lpn_F1	AAACCAGCAGTAGAATCCTGT	21	42.86%	53.25	6423.27	gyrB
Legionella pneumophila	lpn_R1	GGAATTTTCTATCTCGCGCTTG	22	45.45%	54.03	6707.41	gyrB
Legionella pneumophila	lpn_F2	CACTGCAGCAAAATCCATCG	20	50.00%	54.82	6055.02	gyrB
Moraxella	mca_R1	GTTAGCAAACTTAACCGTGA	20	40.00%	49.70	6125.07	oPrI

Microorganism	Primer	Oligonucleotide sequences (5'-3')	bp	GC%	Tm/℃	Molecular Weight	Target gene
catarrhalis							
Moraxella catarrhalis	mca_F2	ACGGAAATCACGACCTGC	18	55.56%	55.60	5477.64	oPrI
Mycoplasma pneumoniae	mpn_F1	ATAACGGTGACTTCTTACCACT	22	40.91%	52.72	6669.41	P1
Mycoplasma pneumoniae	mpn_R1	CGCACTGGTTTGTTTCAAGC	20	50.00%	55.41	6099.02	P1
Mycoplasma pneumoniae	mpn_F2	CCTGATTACGTGTTGCCGTT	20	50.00%	55.65	6090.01	P1
Nocadia asteroides	nas_F1	CACCTACGGCACGAACAACGAG	22	59.09%	61.25	6707.44	secA1
Nocadia asteroides	nas_R2	CTCGGCGTACCACTTGGAC	19	63.16%	59.01	5764.80	secA1
Pseudomonas Aeruginosa	pae_F1	AAGGGGAACACGATGAACAAC	21	47.62%	55.35	6506.33	oPrI
Pseudomonas Aeruginosa	pae_R1	CGAGGGACCGGTTTTCAACA	20	55.00%	58.07	6142.06	oPrI
Pseudomonas Aeruginosa	pae_F2	GCAGCCACTCCAAAGAAACC	20	55.00%	57.22	6049.02	oPrI
Staphylococcus aureus	sau_F2	ATGGCTATCAGTAATGTTTCG	21	38.10%	49.38	6451.27	thermonuclease
Staphylococcus aureus	sau_R2	CTTCTATTTACGCCATTATCTGT	23	34.78%	49.31	6930.57	thermonuclease
Streptococcus	spn_F1	AGTACACGCACACTCAACTGG	21	52.38%	57.52	6384.23	IytA

Microorganism	Primer	Oligonucleotide sequences (5'-3')	bp	GC%	Tm/℃	Molecular Weight	Target gene
pneumoniae							
Streptococcus pneumoniae	spn_R1	TCCCTGTATCAAGCGTTTTCGG	22	50.00%	57.52	6692.40	IytA
Streptococcus pneumoniae	spn_F2	CGGAAAGACCCAGAATTAGGT	21	47.62%	54.20	6488.30	IytA
Aspergillus fumigatus	afu_F1	CCAACATGAACGCTGTTCTGA	21	47.62%	55.53	6390.23	ITS
Aspergillus fumigatus	afu_R1	AGCAGGTGACAAAGCCCCAT	20	55.00%	59.96	6120.06	ITS
Canidida albicans	cal_F2	TCCAACCAGCAAACAATGCG	20	50.00%	56.74	6064.04	3B
Canidida albicans	cal_R1	CTAATGGCAGTTGTAGTAACTCA	23	39.13%	51.50	7062.67	3B
Candida glabrata	cgl_F4	AGGAGGTGTTTTATCACACGA	21	42.86%	53.15	6485.29	ITS
Candida glabrata	cgl_R2	CCCACATACTGATATGGCCTA	21	47.62%	53.38	6350.21	ITS
Candida tropicalis	ctr_F2	TGGGGGCGGGAGCAATC	17	70.59%	62.24	5316.50	ITS
Candida tropicalis	ctr_R1	GCCACTAGCAAAATAAGCGTT	21	42.86%	53.36	6423.27	ITS
Cryptococcus neoformans	cne_F1	GCGAAATGCGATAAGTAATGTGA	23	39.13%	52.95	7160.74	ITS
Cryptococcus neoformans	cne_R2	TAACACATTTAAGGCGAGCCGAC	23	47.83%	57.54	7041.66	ITS
Penicillium marneffei	pma_F1	TGCAGAATTCCGTGAATCATCG	22	45.45%	55.34	6734.45	ITS
Penicillium marneffei	pma_R1	TTACTGAGGCAATCCCGGTT	20	50.00%	56.29	6108.03	ITS
Penicillium	pma_F2	CGTCATTTCTGCCCTCAAGCAC	22	54.55%	59.31	6606.35	ITS

Microorganism	Primer	Oligonucleotide sequences (5’-3’)	bp	GC%	Tm/℃	Molecular Weight	Target gene
marneffei							
Pneumocystis jiroveci	pji_F2	ATACCCGAGAAACACTTAGT	20	40.00%	49.62	6094.06	hypothetical protein
Pneumocystis jiroveci	pji_R1	CGAAATGCCATTAATTCCCAA	21	38.10%	50.68	6358.23	hypothetical protein
human adenoviruses	had_R2	GCCCCAGTGGTCTTACATGCACATC	25	56.00%	62.79	7577.98	hexon protein
human adenoviruses	had_F2	GCCACGGTGGGGTTTCTAAACTT	23	52.17%	60.26	7070.64	hexon protein

Table S2: Microorganisms detected by mNGS and Sanger sequencing in BALF.

No.	mNGS	Sanger sequecing
F01-001	Pneumocystis jirovecii<br>Candida glabrata	Pneumocystis jirovecii<br>Candida glabrata
F01-002	Acinetobacter baumannii	Acinetobacter baumannii
F01-003	Negative	Negative
F01-004	Pneumocystis jirovecii	Pneumocystis jirovecii
F01-005	Negative	Negative
F01-006	Pneumocystis jirovecii<br>Candida albicans<br>Candida tropicalis	Pneumocystis jirovecii<br>Candida albicans<br>Candida tropicalis
F01-007	Legionella pneumophila<br>Pseudomonas aeruginosa	Legionella pneumophila<br>Pseudomonas aeruginosa
F01-008	Klebsiella pneumoniae<br>Acinetobacter baumannii<br>Pseudomonas aeruginosa	Klebsiella pneumoniae<br>Acinetobacter baumannii<br>Pseudomonas aeruginosa
F01-009	Klebsiella pneumoniae<br>Pseudomonas aeruginosa	Klebsiella pneumoniae<br>Pseudomonas aeruginosa
F01-010	Candida albicans<br>Pseudomonas aeruginosa	Candida albicans<br>Pseudomonas aeruginosa
F01-011	Pneumocystis jirovecii<br>Aspergillus fumigatus	Pneumocystis jirovecii<br>Aspergillus fumigatus
F01-012	Pneumocystis jirovecii	Pneumocystis jirovecii
F01-013	Pneumocystis jirovecii	Pneumocystis jirovecii
F01-014	Pneumocystis jirovecii	Pneumocystis jirovecii
F01-015	Negative	Negative
F01-016	Pneumocystis jirovecii	Negative
F01-017	Staphylococcus aureus	Negative
F01-018	Negative	Negative
F01-019	Aspergillus fumigatus<br>Acinetobacter baumannii	Aspergillus fumigatus<br>Acinetobacter baumannii
F01-020	Negative	Pseudomonas aeruginosa
F01-021	Pneumocystis jirovecii	Pneumocystis jirovecii
F01-022	Staphylococcus aureus<br>Aspergillus fumigatus	Staphylococcus aureus<br>Aspergillus fumigatus
F01-023	Negative	Negative
F01-024	Negative	Negative

F01-025	Pneumocystis jirovecii	Pneumocystis jirovecii
F01-026	Negative	Negative
F01-027	Negative	Negative
F01-028	Negative	Negative
F01-029	Negative	Negative
F01-030	Acinetobacter baumannii	Acinetobacter baumannii
F01-031	Candida albicans<br>Candida glabrata	Candida albicans<br>Candida glabrata
F01-032	Klebsiella pneumoniae<br>Candida albicans<br>Acinetobacter baumannii	Klebsiella pneumoniae<br>Candida albicans<br>Acinetobacter baumannii
F01-033	Candida albicans<br>Acinetobacter baumannii	Candida albicans
F01-034	Negative	Negative
F01-035	Candida albicans<br>Cryptococcus neoformans	Candida albicans<br>Cryptococcus neoformans
F01-036	Staphylococcus aureus<br>Pseudomonas aeruginosa	Staphylococcus aureus<br>Pseudomonas aeruginosa
F01-037	Pneumocystis jirovecii	Pneumocystis jirovecii
F01-038	Negative	Negative
F01-039	Pneumocystis jirovecii<br>Acinetobacter baumannii	Pneumocystis jirovecii<br>Acinetobacter baumannii
F01-040	Negative	Negative
F01-041	Pneumocystis jirovecii<br>Legionella pneumophila<br>Klebsiella pneumoniae	Klebsiella pneumoniae<br>Pneumocystis jirovecii
F01-042	Pneumocystis jirovecii	Pneumocystis jirovecii
F01-043	Acinetobacter baumannii	Acinetobacter baumannii
F01-044	Acinetobacter baumannii<br>Pseudomonas aeruginosa	Pseudomonas aeruginosa
F01-045	Pneumocystis jirovecii<br>Candida tropicalis	Pneumocystis jirovecii<br>Candida tropicalis
F01-046	Acinetobacter baumannii	Acinetobacter baumannii

F01-047	Legionella pneumophila<br>Acinetobacter baumannii	Legionella pneumophila<br>Acinetobacter baumannii
F01-048	Candida albicans	Candida albicans
F01-049	Pneumocystis jirovecii<br>Pseudomonas aeruginosa	Pneumocystis jirovecii<br>Pseudomonas aeruginosa
F01-050	Klebsiella pneumoniae<br>Candida albicans<br>Acinetobacter baumannii	Klebsiella pneumoniae<br>Candida albicans<br>Acinetobacter baumannii
F01-051	Aspergillus fumigatus	Aspergillus fumigatus
F01-052	Negative	Negative
F01-053	Aspergillus fumigatus	Aspergillus fumigatus
F01-054	Candida glabrata	Candida glabrata
F01-055	Staphylococcus aureus<br>Acinetobacter baumannii<br>Pseudomonas aeruginosa	Staphylococcus aureus<br>Acinetobacter baumannii<br>Pseudomonas aeruginosa
F01-056	Pneumocystis jirovecii	Pneumocystis jirovecii
F01-057	Klebsiella pneumoniae<br>Pneumocystis jirovecii<br>Pseudomonas aeruginosa	Klebsiella pneumoniae<br>Pneumocystis jirovecii<br>Pseudomonas aeruginosa
F01-058	Negative	Negative
F01-059	Pneumocystis jirovecii<br>Candida albicans<br>Acinetobacter baumannii<br>Pseudomonas aeruginosa	Pneumocystis jirovecii<br>Candida albicans<br>Acinetobacter baumannii<br>Pseudomonas aeruginosa
F01-060	Candida albicans	Candida albicans
F01-061	Pneumocystis jirovecii<br>Aspergillus fumigatus	Pneumocystis jirovecii<br>Aspergillus fumigatus
F01-062	Klebsiella pneumoniae	Klebsiella pneumoniae
F01-063	Pneumocystis jirovecii<br>Candida albicans<br>Acinetobacter baumannii	Pneumocystis jirovecii<br>Candida albicans<br>Acinetobacter baumannii
F01-064	Klebsiella pneumoniae	Klebsiella pneumoniae
F01-065	Acinetobacter baumannii	Acinetobacter baumannii

F01-066	Negative	Negative
F01-067	Legionella pneumophila	Legionella pneumophila
F01-068	Pneumocystis jirovecii<br>Acinetobacter baumannii<br>Pseudomonas aeruginosa	Acinetobacter baumannii<br>Pseudomonas aeruginosa
F01-069	Pneumocystis jirovecii<br>Acinetobacter baumannii	Pneumocystis jirovecii<br>Acinetobacter baumannii
F01-070	Pneumocystis jirovecii	Pneumocystis jirovecii
F01-071	Negative	Negative
F01-072	Negative	Negative
F01-073	Pneumocystis jirovecii<br>Acinetobacter baumannii<br>Pseudomonas aeruginosa	Pneumocystis jirovecii<br>Acinetobacter baumannii
F01-074	Klebsiella pneumoniae<br>Pneumocystis jirovecii	Klebsiella pneumoniae<br>Pneumocystis jirovecii
F01-075	Candida albicans<br>Acinetobacter baumannii	Candida albicans<br>Acinetobacter baumannii
F01-076	Negative	Negative
F01-077	Pneumocystis jirovecii<br>Aspergillus fumigatus	Pneumocystis jirovecii<br>Aspergillus fumigatus
F01-078	Aspergillus fumigatus	Negative
F01-079	Candida albicans<br>Candida tropicalis<br>Acinetobacter baumannii<br>Pseudomonas aeruginosa	Candida albicans<br>Candida tropicalis<br>Acinetobacter baumannii<br>Pseudomonas aeruginosa
F01-080	Negative	Negative
F01-081	Negative	Negative
F01-082	Pneumocystis jirovecii	Pneumocystis jirovecii
F01-083	Candida albicans	Candida albicans
F01-084	Klebsiella pneumoniae	Negative
F01-085	Candida albicans<br>Pseudomonas aeruginosa	Candida albicans<br>Pseudomonas aeruginosa

F01-086	Klebsiella pneumoniae<br>Acinetobacter baumannii<br>Pseudomonas aeruginosa	Klebsiella pneumoniae<br>Acinetobacter baumannii<br>Pseudomonas aeruginosa
F01-087	Candida albicans<br>Staphylococcus aureus	Candida albicans<br>Staphylococcus aureus
F01-088	Acinetobacter baumannii	Candida albicans<br>Acinetobacter baumannii
F01-089	Pneumocystis jirovecii<br>Staphylococcus aureus	Pneumocystis jirovecii<br>Staphylococcus aureus
F01-090	Acinetobacter baumannii	Acinetobacter baumannii
F01-091	Acinetobacter baumannii	Acinetobacter baumannii
F01-092	Aspergillus fumigatus<br>Acinetobacter baumannii	Aspergillus fumigatus<br>Acinetobacter baumannii
F01-093	Staphylococcus aureus<br>Acinetobacter baumannii	Staphylococcus aureus<br>Acinetobacter baumannii
F01-094	Klebsiella pneumoniae<br>Pneumocystis jirovecii<br>Aspergillus fumigatus<br>Pseudomonas aeruginosa	Klebsiella pneumoniae<br>Pneumocystis jirovecii<br>Aspergillus fumigatus<br>Pseudomonas aeruginosa
F01-095	Klebsiella pneumoniae<br>Staphylococcus aureus	Klebsiella pneumoniae<br>Staphylococcus aureus
F01-096	Acinetobacter baumannii	Acinetobacter baumannii
F01-097	Staphylococcus aureus	Staphylococcus aureus
F01-098	Acinetobacter baumannii<br>Pseudomonas aeruginosa	Acinetobacter baumannii<br>Pseudomonas aeruginosa
F01-099	Pneumocystis jirovecii	Pneumocystis jirovecii
F01-100	Pneumocystis jirovecii<br>Candida tropicalis	Pneumocystis jirovecii<br>Candida tropicalis
F01-101	Acinetobacter baumannii	Acinetobacter baumannii
F01-102	Negative	Negative
F01-103	Negative	Negative
F01-104	Acinetobacter baumannii	Acinetobacter baumannii
F01-105	Candida albicans	Candida albicans

F01-106	Candida albicans<br>Staphylococcus aureus<br>Pseudomonas aeruginosa	Candida albicans<br>Staphylococcus aureus<br>Pseudomonas aeruginosa
F01-107	Pneumocystis jirovecii<br>Aspergillus fumigatus	Pneumocystis jirovecii<br>Aspergillus fumigatus
F01-108	Klebsiella pneumoniae<br>Pneumocystis jirovecii<br>Candida albicans	Klebsiella pneumoniae<br>Pneumocystis jirovecii<br>Candida albicans
F01-109	Negative	Negative
F01-110	Negative	Negative
F01-111	Acinetobacter baumannii	Acinetobacter baumannii
F01-112	Negative	Negative
F01-113	Negative	Negative
F01-114	Negative	Negative
F01-115	Negative	Negative
F01-116	Acinetobacter baumannii	Acinetobacter baumannii
F01-117	Negative	Negative
F01-118	Acinetobacter baumannii	Acinetobacter baumannii
F01-119	Candida glabrata	Candida glabrata
F01-120	Negative	Negative
F01-121	Negative	Negative
F01-122	Klebsiella pneumoniae<br>Acinetobacter baumannii<br>Pseudomonas aeruginosa	Klebsiella pneumoniae<br>Pseudomonas aeruginosa
F01-123	Aspergillus fumigatus	Aspergillus fumigatus
F01-124	Negative	Negative
F01-125	Acinetobacter baumannii	Acinetobacter baumannii
F01-126	Pneumocystis jirovecii	Pneumocystis jirovecii
F01-127	Candida albicans	Candida albicans
F01-128	Streptococcus pneumoniae<br>Aspergillus fumigatus	Streptococcus pneumoniae<br>Aspergillus fumigatus
F01-129	Pneumocystis jirovecii	Pneumocystis jirovecii

F01-130	Aspergillus fumigatus<br>Acinetobacter baumannii	Aspergillus fumigatus<br>Acinetobacter baumannii
F01-131	Candida albicans	Negative
F01-132	Aspergillus fumigatus	Aspergillus fumigatus
F01-133	Legionella pneumophila	Legionella pneumophila
F01-134	Pneumocystis jirovecii<br>Aspergillus fumigatus	Pneumocystis jirovecii<br>Aspergillus fumigatus
F01-135	Negative	Negative
F01-136	Aspergillus fumigatus<br>Pneumocystis jirovecii<br>Candida albicans	Pneumocystis jirovecii<br>Aspergillus fumigatus
F01-137	Klebsiella pneumoniae<br>Pseudomonas aeruginosa	Klebsiella pneumoniae<br>Pseudomonas aeruginosa
F01-138	Negative	Negative
F01-139	Negative	Negative
F01-140	Acinetobacter baumannii	Acinetobacter baumannii
F01-141	Negative	Negative
F01-142	Staphylococcus aureus	Staphylococcus aureus
F01-143	Negative	Negative
F01-144	Pseudomonas aeruginosa	Pseudomonas aeruginosa
F01-145	Negative	Negative
F01-146	Negative	Negative
F01-147	Negative	Negative
F01-148	Negative	Negative
F01-149	Negative	Negative
F01-150	Legionella pneumophila	Legionella pneumophila
F01-151	Aspergillus fumigatus<br>Acinetobacter baumannii<br>Pseudomonas aeruginosa	Aspergillus fumigatus<br>Acinetobacter baumannii<br>Pseudomonas aeruginosa
F01-152	Negative	Negative
F01-153	Klebsiella pneumoniae<br>Candida glabrata<br>Acinetobacter baumannii	Klebsiella pneumoniae<br>Candida glabrata<br>Acinetobacter baumannii

F01-154	Candida albicans<br>Pseudomonas aeruginosa	Candida albicans<br>Pseudomonas aeruginosa
F01-155	Candida albicans	Candida albicans
F01-156	Negative	Negative
F01-157	Acinetobacter baumannii	Negative
F01-158	Candida albicans<br>Acinetobacter baumannii	Candida albicans<br>Acinetobacter baumannii
F01-159	Negative	Negative
F01-160	Klebsiella pneumoniae<br>Candida albicans	Klebsiella pneumoniae<br>Candida albicans
F01-161	Pneumocystis jirovecii<br>Acinetobacter baumannii<br>Pseudomonas aeruginosa	Pneumocystis jirovecii<br>Acinetobacter baumannii<br>Pseudomonas aeruginosa
F01-162	Negative	Negative
F01-163	Pneumocystis jirovecii<br>Candida albicans	Pneumocystis jirovecii<br>Candida albicans
F01-164	Negative	Negative
F01-165	Negative	Negative
F01-166	Negative	Negative
F01-167	Acinetobacter baumannii<br>Pseudomonas aeruginosa	Acinetobacter baumannii
F01-168	Pneumocystis jirovecii	Pneumocystis jirovecii
F01-169	Candida albicans<br>Acinetobacter baumannii	Candida albicans<br>Acinetobacter baumannii
F01-170	Negative	Negative
F01-171	Negative	Negative
F01-172	Staphylococcus aureus<br>Pseudomonas aeruginosa	Staphylococcus aureus<br>Pseudomonas aeruginosa
F01-173	Klebsiella pneumoniae	Klebsiella pneumoniae
F01-174	Candida albicans<br>Pseudomonas aeruginosa	Candida albicans<br>Pseudomonas aeruginosa
F01-175	Acinetobacter baumannii	Acinetobacter baumannii
F01-176	Staphylococcus aureus	Staphylococcus aureus

F01-177	Klebsiella pneumoniae<br>Candida glabrata	Klebsiella pneumoniae<br>Candida glabrata
F01-178	Candida albicans<br>Acinetobacter baumannii	Candida albicans<br>Acinetobacter baumannii
F01-179	Negative	Negative
F01-180	Candida tropicalis	Candida tropicalis
F01-181	Pneumocystis jirovecii	Pneumocystis jirovecii
F01-182	Negative	Negative
F01-183	Klebsiella pneumoniae	Klebsiella pneumoniae
F01-184	Candida albicans<br>Acinetobacter baumannii	Candida albicans<br>Acinetobacter baumannii

Table S3. Microorganisms detected by mNGS and Sanger sequencing in Sputum.

No.	mNGS	Sanger sequecing
T01-001	Pseudomonas aeruginosa	Pseudomonas aeruginosa
T01-002	Haemophilus influenzae	Haemophilus influenzae
T01-003	Aspergillus fumigatus	Aspergillus fumigatus
T01-004	Pseudomonas aeruginosa	Pseudomonas aeruginosa
T01-005	Candida albicans<br>Acinetobacter baumannii<br>Staphylococcus aureus	Candida albicans<br>Acinetobacter baumannii
T01-006	Pneumocystis jirovecii	Pneumocystis jirovecii
T01-007	Candida albicans	Candida albicans
T01-008	Aspergillus fumigatus<br>Haemophilus influenzae<br>Staphylococcus aureus	Haemophilus influenzae<br>Staphylococcus aureus<br>Aspergillus fumigatus
T01-009	Candida albicans<br>Streptococcus pneumoniae<br>Pseudomonas aeruginosa	Candida albicans<br>Pseudomonas aeruginosa
T01-010	Klebsiella pneumoniae	Klebsiella pneumoniae
T01-011	Pneumocystis jirovecii<br>Candida albicans<br>Streptococcus pneumoniae	Pneumocystis jirovecii<br>Candida albicans<br>Streptococcus pneumoniae
T01-012	Haemophilus influenzae<br>Moraxella catarrhalis	Moraxella catarrhalis
T01-013	Haemophilus influenzae<br>Pseudomonas aeruginosa	Haemophilus influenzae<br>Pseudomonas aeruginosa
T01-014	Candida albicans<br>Candida glabrata<br>Streptococcus pneumoniae	Candida albicans<br>Streptococcus pneumoniae<br>Candida glabrata
T01-015	Legionella pneumophila<br>Pseudomonas aeruginosa	Legionella pneumophila<br>Pseudomonas aeruginosa
T01-016	Moraxella catarrhalis<br>Klebsiella pneumoniae	Klebsiella pneumoniae<br>Moraxella catarrhalis
T01-017	Aspergillus fumigatus<br>Acinetobacter baumannii	Aspergillus fumigatus<br>Acinetobacter baumannii
T01-018	Pneumocystis jirovecii<br>Candida tropicalis<br>Candida glabrata	Pneumocystis jirovecii<br>Candida tropicalis<br>Candida glabrata
T01-019	Candida albicans<br>Candida glabrata	Candida glabrata
T01-020	Haemophilus influenzae<br>Moraxella catarrhalis<br>Staphylococcus aureus	Haemophilus influenzae<br>Staphylococcus aureus<br>Moraxella catarrhalis

T01-021	Pneumocystis jirovecii<br>Candida albicans<br>Candida tropicalis<br>Klebsiella pneumoniae<br>Acinetobacter baumannii	Klebsiella pneumoniae<br>Pneumocystis jirovecii<br>Candida albicans<br>Candida tropicalis
T01-022	Pneumocystis jirovecii	Pneumocystis jirovecii
T01-023	Pneumocystis jirovecii<br>Candida albicans	Pneumocystis jirovecii<br>Candida albicans
T01-024	Pneumocystis jirovecii<br>Candida albicans<br>Streptococcus pneumoniae	Candida albicans<br>Streptococcus pneumoniae
T01-025	Haemophilus influenzae	Haemophilus influenzae
T01-026	Haemophilus influenzae	Haemophilus influenzae
T01-027	Pneumocystis jirovecii	Pneumocystis jirovecii
T01-028	Haemophilus influenzae	Haemophilus influenzae
T01-029	Candida tropicalis	Candida tropicalis
T01-030	Haemophilus influenzae<br>Moraxella catarrhalis	Haemophilus influenzae<br>Moraxella catarrhalis
T01-031	Aspergillus fumigatus<br>Candida tropicalis<br>Streptococcus pneumoniae<br>Klebsiella pneumoniae<br>Acinetobacter baumannii	Streptococcus pneumoniae<br>Candida tropicalis<br>Aspergillus fumigatus<br>Acinetobacter baumannii

T01-032	Aspergillus fumigatus<br>Pneumocystis jirovecii<br>Candida albicans<br>Pseudomonas aeruginosa	Pneumocystis jirovecii<br>Candida albicans<br>Aspergillus fumigatus<br>Pseudomonas aeruginosa
T01-033	Aspergillus fumigatus<br>Candida albicans<br>Klebsiella pneumoniae<br>Pseudomonas aeruginosa<br>Staphylococcus aureus	Klebsiella pneumoniae<br>Candida albicans<br>Aspergillus fumigatus<br>Pseudomonas aeruginosa
T01-034	Streptococcus pneumoniae<br>Pseudomonas aeruginosa	Streptococcus pneumoniae<br>Pseudomonas aeruginosa
T01-035	Staphylococcus aureus	Staphylococcus aureus
T01-036	Haemophilus influenzae	Haemophilus influenzae
T01-037	Candida tropicalis<br>Candida glabrata<br>Streptococcus pneumoniae<br>Klebsiella pneumoniae<br>Pseudomonas aeruginosa	Klebsiella pneumoniae<br>Streptococcus pneumoniae<br>Candida tropicalis<br>Candida glabrata<br>Pseudomonas aeruginosa
T01-038	Aspergillus fumigatus	Aspergillus fumigatus
T01-039	Candida albicans<br>Haemophilus influenzae	Candida albicans<br>Haemophilus influenzae
T01-040	Haemophilus influenzae	Haemophilus influenzae

T01-041	Pneumocystis jirovecii<br>Acinetobacter baumannii	Pneumocystis jirovecii<br>Acinetobacter baumannii
T01-042	Pneumocystis jirovecii<br>Candida tropicalis	Pneumocystis jirovecii<br>Candida tropicalis<br>Acinetobacter baumannii
T01-043	Candida albicans<br>Candida glabrata<br>Pseudomonas aeruginosa	Candida albicans<br>Candida glabrata<br>Pseudomonas aeruginosa
T01-044	Aspergillus fumigatus<br>Staphylococcus aureus	Staphylococcus aureus<br>Aspergillus fumigatus
T01-045	Pneumocystis jirovecii<br>Candida albicans	Pneumocystis jirovecii<br>Candida albicans
T01-046	Aspergillus fumigatus<br>Pneumocystis jirovecii<br>Candida albicans<br>Streptococcus pneumoniae<br>Moraxella catarrhalis	Pneumocystis jirovecii<br>Candida albicans<br>Aspergillus fumigatus
T01-047	Candida albicans<br>Candida tropicalis<br>Legionella pneumophila	Legionella pneumophila<br>Candida albicans<br>Candida tropicalis
T01-048	Haemophilus influenzae	Haemophilus influenzae
T01-049	Streptococcus pneumoniae	Streptococcus pneumoniae
T01-050	Candida albicans<br>Moraxella catarrhalis	Candida albicans<br>Moraxella catarrhalis
T01-051	Streptococcus pneumoniae<br>Moraxella catarrhalis	Streptococcus pneumoniae<br>Moraxella catarrhalis

T01-052	Candida albicans<br>Klebsiella pneumoniae<br>Acinetobacter baumannii	Klebsiella pneumoniae<br>Candida albicans<br>Acinetobacter baumannii
T01-053	Candida albicans	Candida albicans
T01-054	Candida albicans<br>Staphylococcus aureus	Candida albicans<br>Staphylococcus aureus
T01-055	Candida albicans	Candida albicans
T01-056	Moraxella catarrhalis	Moraxella catarrhalis
T01-057	Aspergillus fumigatus<br>Pneumocystis jirovecii<br>Candida albicans<br>Candida tropicalis<br>Klebsiella pneumoniae	Klebsiella pneumoniae<br>Pneumocystis jirovecii<br>Candida albicans<br>Candida tropicalis<br>Aspergillus fumigatus
T01-058	Candida albicans<br>Candida glabrata	Candida albicans<br>Candida glabrata
T01-059	Pneumocystis jirovecii	Pneumocystis jirovecii
T01-060	Aspergillus fumigatus<br>Candida albicans<br>Streptococcus pneumoniae	Candida albicans<br>Streptococcus pneumoniae
T01-061	Pneumocystis jirovecii<br>Candida albicans	Pneumocystis jirovecii<br>Candida albicans
T01-062	Staphylococcus aureus	Staphylococcus aureus
T01-063	Aspergillus fumigatus	Aspergillus fumigatus

T01-064	Aspergillus fumigatus<br>Pneumocystis jirovecii	Pneumocystis jirovecii<br>Aspergillus fumigatus
T01-065	Candida tropicalis	Candida tropicalis
T01-066	Aspergillus fumigatus<br>Haemophilus influenzae	Haemophilus influenzae<br>Aspergillus fumigatus
T01-067	Aspergillus fumigatus<br>Pneumocystis jirovecii<br>Pseudomonas aeruginosa	Pneumocystis jirovecii<br>Pseudomonas aeruginosa
T01-068	Haemophilus influenzae	Haemophilus influenzae
T01-069	Candida albicans<br>Candida glabrata<br>Klebsiella pneumoniae<br>Pseudomonas aeruginosa<br>Staphylococcus aureus	Klebsiella pneumoniae<br>Candida albicans<br>Staphylococcus aureus<br>Candida glabrata<br>Pseudomonas aeruginosa
T01-070	Candida albicans<br>Candida tropicalis<br>Staphylococcus aureus	Candida albicans<br>Staphylococcus aureus<br>Candida tropicalis
T01-071	Aspergillus fumigatus<br>Haemophilus influenzae<br>Pseudomonas aeruginosa	Aspergillus fumigatus<br>Pseudomonas aeruginosa
T01-072	Candida albicans<br>Staphylococcus aureus	Candida albicans<br>Staphylococcus aureus

T01-073	Pneumocystis jirovecii<br>Candida albicans<br>Klebsiella pneumoniae<br>Staphylococcus aureus	Klebsiella pneumoniae<br>Candida albicans<br>Staphylococcus aureus
T01-074	Haemophilus influenzae	Haemophilus influenzae
T01-075	Candida albicans<br>Candida glabrata	Candida albicans<br>Candida glabrata
T01-076	Acinetobacter baumannii<br>Staphylococcus aureus	Staphylococcus aureus<br>Acinetobacter baumannii
T01-077	Aspergillus fumigatus<br>Klebsiella pneumoniae<br>Pseudomonas aeruginosa	Klebsiella pneumoniae<br>Aspergillus fumigatus<br>Pseudomonas aeruginosa
T01-078	Candida glabrata	Candida glabrata
T01-079	Pneumocystis jirovecii<br>Candida albicans<br>Candida tropicalis	Pneumocystis jirovecii<br>Candida albicans<br>Candida tropicalis
T01-080	Candida albicans<br>Staphylococcus aureus	Candida albicans<br>Staphylococcus aureus
T01-081	Aspergillus fumigatus<br>Pneumocystis jirovecii<br>Candida albicans	Pneumocystis jirovecii<br>Candida albicans<br>Aspergillus fumigatus

T01-082	Candida albicans<br>Candida glabrata<br>Streptococcus pneumoniae<br>Klebsiella pneumoniae<br>Pseudomonas aeruginosa	Klebsiella pneumoniae<br>Candida albicans<br>Streptococcus pneumoniae<br>Candida glabrata<br>Pseudomonas aeruginosa
T01-083	Pseudomonas aeruginosa	Pseudomonas aeruginosa
T01-084	Klebsiella pneumoniae	Klebsiella pneumoniae
T01-085	Acinetobacter baumannii<br>Pseudomonas aeruginosa<br>Staphylococcus aureus	Staphylococcus aureus<br>Acinetobacter baumannii<br>Pseudomonas aeruginosa
T01-086	Candida albicans<br>Haemophilus influenzae<br>Moraxella catarrhalis	Candida albicans<br>Haemophilus influenzae<br>Moraxella catarrhalis
T01-087	Candida tropicalis<br>Streptococcus pneumoniae	Streptococcus pneumoniae<br>Candida tropicalis
T01-088	Candida albicans<br>Candida glabrata<br>Moraxella catarrhalis	Candida albicans<br>Candida glabrata<br>Moraxella catarrhalis
T01-089	Pseudomonas aeruginosa<br>Staphylococcus aureus	Staphylococcus aureus<br>Pseudomonas aeruginosa
T01-090	Klebsiella pneumoniae<br>Acinetobacter baumannii	Klebsiella pneumoniae<br>Acinetobacter baumannii

T01-091	Klebsiella pneumoniae<br>Pseudomonas aeruginosa	Klebsiella pneumoniae<br>Pseudomonas aeruginosa
T01-092	Candida tropicalis	Candida tropicalis
T01-093	Klebsiella pneumoniae	Klebsiella pneumoniae
T01-094	Pseudomonas aeruginosa	Pseudomonas aeruginosa
T01-095	Candida albicans<br>Candida glabrata	Candida albicans<br>Candida glabrata
T01-096	Acinetobacter baumannii<br>Pseudomonas aeruginosa	Acinetobacter baumannii<br>Pseudomonas aeruginosa
T01-097	Streptococcus pneumoniae<br>Staphylococcus aureus	Staphylococcus aureus<br>Streptococcus pneumoniae
T01-098	Staphylococcus aureus	Staphylococcus aureus
T01-099	Aspergillus fumigatus<br>Streptococcus pneumoniae	Streptococcus pneumoniae<br>Aspergillus fumigatus
T01-100	Acinetobacter baumannii<br>Pseudomonas aeruginosa	Acinetobacter baumannii<br>Pseudomonas aeruginosa
T01-101	Candida albicans	Candida albicans<br>Pseudomonas aeruginosa
T01-102	Candida albicans<br>Staphylococcus aureus	Candida albicans<br>Staphylococcus aureus
T01-103	Candida albicans	Candida albicans

T01-104	Pseudomonas aeruginosa	Pseudomonas aeruginosa
T01-105	Candida albicans<br>Staphylococcus aureus<br>Acinetobacter baumannii	Candida albicans<br>Staphylococcus aureus<br>Acinetobacter baumannii
T01-106	Aspergillus fumigatus<br>Acinetobacter baumannii	Aspergillus fumigatus<br>Acinetobacter baumannii
T01-107	Candida albicans<br>Pseudomonas aeruginosa<br>Cryptococcus neoformans	Candida albicans<br>Pseudomonas aeruginosa<br>Cryptococcus neoformans
T01-108	Acinetobacter baumannii	Acinetobacter baumannii
T01-109	Negative	Candida albicans
T01-110	human adenoviruses	human adenoviruses
T01-111	Candida albicans<br>Candida glabrata<br>Aspergillus fumigatus<br>Pseudomonas aeruginosa	Candida albicans<br>Candida glabrata<br>Aspergillus fumigatus<br>Pseudomonas aeruginosa
T01-112	Acinetobacter baumannii	Acinetobacter baumannii
T01-113	Klebsiella pneumoniae	Klebsiella pneumoniae
T01-114	Candida albicans<br>Candida glabrata<br>Acinetobacter baumannii	Candida albicans<br>Candida glabrata<br>Acinetobacter baumannii

T01-115	Klebsiella pneumoniae<br>Candida albicans<br>Candida tropicalis<br>Acinetobacter baumannii	Klebsiella pneumoniae<br>Candida albicans<br>Candida tropicalis<br>Acinetobacter baumannii
T01-116	Pneumocystis jirovecii	Pneumocystis jirovecii
T01-117	Aspergillus fumigatus<br>Acinetobacter baumannii	Aspergillus fumigatus<br>Acinetobacter baumannii
T01-118	Klebsiella pneumoniae	Klebsiella pneumoniae
T01-119	Candida tropicalis<br>Pseudomonas aeruginosa	Candida tropicalis<br>Pseudomonas aeruginosa
T01-120	Candida albicans<br>Candida glabrata<br>Klebsiella pneumoniae<br>Staphylococcus aureus	Candida albicans<br>Staphylococcus aureus<br>Candida glabrata
T01-121	Aspergillus fumigatus<br>Pneumocystis jirovecii<br>Pseudomonas aeruginosa<br>Staphylococcus aureus	Aspergillus fumigatus<br>Pseudomonas aeruginosa
T01-122	Candida albicans<br>Pseudomonas aeruginosa	Candida albicans<br>Pseudomonas aeruginosa
T01-123	Aspergillus fumigatus	Aspergillus fumigatus
T01-124	Staphylococcus aureus<br>Acinetobacter baumannii<br>Pseudomonas aeruginosa	Staphylococcus aureus<br>Acinetobacter baumannii<br>Pseudomonas aeruginosa
T01-125	Candida glabrata<br>Klebsiella pneumoniae	Candida glabrata<br>human adenoviruses

T01-126	Klebsiella pneumoniae<br>Candida albicans	Klebsiella pneumoniae<br>Candida albicans
T01-127	Candida albicans	Candida albicans
T01-128	Acinetobacter baumannii<br>human adenoviruses	Acinetobacter baumannii<br>human adenoviruses
T01-129	Klebsiella pneumoniae<br>Pseudomonas aeruginosa	Klebsiella pneumoniae<br>Pseudomonas aeruginosa
T01-130	Candida albicans	Candida albicans
T01-131	Candida albicans<br>Cryptococcus neoformans	Candida albicans<br>Cryptococcus neoformans
T01-132	Klebsiella pneumoniae<br>Aspergillus fumigatus<br>Acinetobacter baumannii	Klebsiella pneumoniae<br>Aspergillus fumigatus<br>Acinetobacter baumannii
T01-133	Candida albicans<br>Candida glabrata<br>Acinetobacter baumannii	Candida albicans<br>Candida glabrata
T01-134	Pneumocystis jirovecii<br>Pseudomonas aeruginosa	Pneumocystis jirovecii<br>Pseudomonas aeruginosa
T01-135	Staphylococcus aureus<br>Acinetobacter baumannii<br>Pseudomonas aeruginosa	Staphylococcus aureus<br>Acinetobacter baumannii<br>Pseudomonas aeruginosa

T01-136	Pneumocystis jirovecii<br>Staphylococcus aureus<br>Acinetobacter baumannii	Pneumocystis jirovecii<br>Staphylococcus aureus<br>Acinetobacter baumannii
T01-137	Candida albicans<br>Staphylococcus aureus	Candida albicans
T01-138	Klebsiella pneumoniae	Klebsiella pneumoniae
T01-139	Cryptococcus neoformans	Cryptococcus neoformans
T01-140	Negative	Negative
T01-141	Negative	Negative
T01-142	Klebsiella pneumoniae	Klebsiella pneumoniae
T01-143	Streptococcus pneumoniae	Streptococcus pneumoniae
T01-144	Haemophilus influenzae	Haemophilus influenzae
T01-145	Klebsiella pneumoniae<br>Acinetobacter baumannii	Klebsiella pneumoniae<br>Acinetobacter baumannii
T01-146	Aspergillus fumigatus	Aspergillus fumigatus
T01-147	Acinetobacter baumannii	Acinetobacter baumannii
T01-148	Pneumocystis jirovecii<br>Staphylococcus aureus	Pneumocystis jirovecii<br>Staphylococcus aureus
T01-149	Pneumocystis jirovecii<br>Candida albicans	Pneumocystis jirovecii<br>Candida albicans
T01-150	Acinetobacter baumannii	Acinetobacter baumannii

T01-151	Aspergillus fumigatus	Aspergillus fumigatus
T01-152	Aspergillus fumigatus<br>Candida albicans<br>Candida tropicalis<br>Klebsiella pneumoniae<br>Acinetobacter baumannii<br>Pseudomonas aeruginosa<br>Staphylococcus aureus	Klebsiella pneumoniae<br>Candida albicans<br>Candida tropicalis<br>Aspergillus fumigatus<br>Acinetobacter baumannii
T01-153	Staphylococcus aureus	Staphylococcus aureus
T01-154	Candida albicans<br>Haemophilus influenzae<br>Staphylococcus aureus	Candida albicans<br>Haemophilus influenzae<br>Staphylococcus aureus
T01-155	Haemophilus influenzae<br>Staphylococcus aureus	Haemophilus influenzae<br>Staphylococcus aureus
T01-156	Acinetobacter baumannii<br>Pseudomonas aeruginosa	Acinetobacter baumannii<br>Pseudomonas aeruginosa
T01-157	Candida albicans<br>Pseudomonas aeruginosa	Candida albicans<br>Pseudomonas aeruginosa
T01-158	Negative	Negative
T01-159	Klebsiella pneumoniae<br>Aspergillus fumigatus	Klebsiella pneumoniae<br>Aspergillus fumigatus
T01-160	Candida albicans<br>Acinetobacter baumannii	Candida albicans<br>Acinetobacter baumannii

T01-161	Staphylococcus aureus	Klebsiella pneumoniae<br>Staphylococcus aureus
T01-162	Candida albicans<br>Staphylococcus aureus<br>Candida glabrata	Candida albicans<br>Staphylococcus aureus<br>Candida glabrata
T01-163	Acinetobacter baumannii	Acinetobacter baumannii
T01-164	Klebsiella pneumoniae<br>Staphylococcus aureus<br>Pseudomonas aeruginosa	Klebsiella pneumoniae<br>Staphylococcus aureus<br>Pseudomonas aeruginosa
T01-165	Candida albicans<br>Aspergillus fumigatus<br>Acinetobacter baumannii	Candida albicans<br>Aspergillus fumigatus<br>Acinetobacter baumannii
T01-166	Klebsiella pneumoniae	Klebsiella pneumoniae
T01-167	Haemophilus influenzae<br>Streptococcus pneumoniae<br>Moraxella catarrhalis	Haemophilus influenzae<br>Streptococcus pneumoniae<br>Moraxella catarrhalis
T01-168	Candida tropicalis	Candida tropicalis
T01-169	Moraxella catarrhalis	Moraxella catarrhalis
T01-170	Negative	Negative
T01-171	Aspergillus fumigatus	Aspergillus fumigatus

T01-172	Pneumocystis jirovecii<br>Staphylococcus aureus<br>Pseudomonas aeruginosa<br>Cryptococcus neoformans<br>Candida albicans	Pneumocystis jirovecii<br>Staphylococcus aureus<br>Pseudomonas aeruginosa<br>Cryptococcus neoformans
T01-173	Pneumocystis jirovecii<br>Streptococcus pneumoniae<br>Aspergillus fumigatus	Pneumocystis jirovecii<br>Streptococcus pneumoniae<br>Aspergillus fumigatus
T01-174	Klebsiella pneumoniae<br>Pneumocystis jirovecii<br>Staphylococcus aureus	Klebsiella pneumoniae<br>Pneumocystis jirovecii<br>Staphylococcus aureus
T01-175	Acinetobacter baumannii	Acinetobacter baumannii
T01-176	Candida albicans<br>Staphylococcus aureus<br>Acinetobacter baumannii<br>Pseudomonas aeruginosa	Candida albicans<br>Staphylococcus aureus<br>Acinetobacter baumannii<br>Pseudomonas aeruginosa
T01-177	Klebsiella pneumoniae<br>Staphylococcus aureus<br>Candida glabrata<br>Acinetobacter baumannii	Klebsiella pneumoniae<br>Staphylococcus aureus<br>Candida glabrata<br>Acinetobacter baumannii

T01-178	Staphylococcus aureus<br>Streptococcus pneumoniae<br>Candida tropicalis<br>Candida glabrata<br>Acinetobacter baumannii	Staphylococcus aureus<br>Streptococcus pneumoniae<br>Candida tropicalis<br>Candida glabrata<br>Acinetobacter baumannii
T01-179	Klebsiella pneumoniae<br>Acinetobacter baumannii<br>Pseudomonas aeruginosa	Klebsiella pneumoniae<br>Pseudomonas aeruginosa
T01-180	Candida albicans<br>Staphylococcus aureus	Candida albicans
T01-181	Candida albicans<br>Candida glabrata	Candida albicans<br>Candida glabrata
T01-182	Pseudomonas aeruginosa	Pseudomonas aeruginosa
T01-183	Haemophilus influenzae	Haemophilus influenzae
T01-184	Candida glabrata	Candida glabrata
T01-185	Candida tropicalis	Candida tropicalis
T01-186	Staphylococcus aureus	Staphylococcus aureus
T01-187	Staphylococcus aureus	Staphylococcus aureus
T01-188	Negative	Negative
T01-189	Staphylococcus aureus	Staphylococcus aureus
T01-190	Pseudomonas aeruginosa	Pseudomonas aeruginosa
T01-191	Haemophilus influenzae<br>Staphylococcus aureus	Haemophilus influenzae<br>Staphylococcus aureus
T01-192	Haemophilus influenzae	Haemophilus influenzae

T01-193	Klebsiella pneumoniae<br>Pneumocystis jirovecii<br>Streptococcus pneumoniae<br>Candida glabrata<br>Acinetobacter baumannii	Klebsiella pneumoniae<br>Pneumocystis jirovecii<br>Streptococcus pneumoniae<br>Candida glabrata<br>Acinetobacter baumannii
T01-194	Klebsiella pneumoniae<br>Candida albicans<br>Haemophilus influenzae<br>Staphylococcus aureus	Klebsiella pneumoniae<br>Candida albicans<br>Haemophilus influenzae<br>Staphylococcus aureus
T01-195	Candida glabrata	Candida glabrata
T01-196	Pseudomonas aeruginosa	Pseudomonas aeruginosa
T01-197	Klebsiella pneumoniae<br>Acinetobacter baumannii	Klebsiella pneumoniae<br>Acinetobacter baumannii
T01-198	Staphylococcus aureus<br>Pseudomonas aeruginosa	Staphylococcus aureus<br>Pseudomonas aeruginosa
T01-199	Candida albicans<br>Staphylococcus aureus<br>Candida glabrata<br>Acinetobacter baumannii	Candida albicans<br>Staphylococcus aureus<br>Candida glabrata<br>Acinetobacter baumannii
T01-200	Candida glabrata<br>Acinetobacter baumannii<br>human adenoviruses	Candida glabrata<br>Acinetobacter baumannii
T01-201	Klebsiella pneumoniae<br>Candida albicans<br>Acinetobacter baumannii	Klebsiella pneumoniae<br>Candida albicans<br>Acinetobacter baumannii
T01-202	Staphylococcus aureus	Staphylococcus aureus

T01-203	Klebsiella pneumoniae<br>Acinetobacter baumannii<br>Staphylococcus aureus	Klebsiella pneumoniae<br>Staphylococcus aureus
T01-204	Staphylococcus aureus	Staphylococcus aureus
T01-205	Klebsiella pneumoniae<br>Candida albicans<br>Staphylococcus aureus	Klebsiella pneumoniae<br>Candida albicans<br>Staphylococcus aureus
T01-206	Pseudomonas aeruginosa	Pseudomonas aeruginosa
T01-207	Negative	Negative
T01-208	Klebsiella pneumoniae<br>Acinetobacter baumannii	Klebsiella pneumoniae<br>Acinetobacter baumannii
T01-209	Candida albicans	Candida albicans
T01-210	Aspergillus fumigatus<br>Staphylococcus aureus	Staphylococcus aureus<br>Aspergillus fumigatus
T01-211	Candida albicans<br>Acinetobacter baumannii	Candida albicans<br>Aspergillus fumigatus<br>Acinetobacter baumannii
T01-212	Klebsiella pneumoniae<br>Pseudomonas aeruginosa	Klebsiella pneumoniae<br>Pseudomonas aeruginosa
T01-213	Klebsiella pneumoniae<br>Pseudomonas aeruginosa	Klebsiella pneumoniae<br>Pseudomonas aeruginosa
T01-214	Candida albicans	Candida albicans

T01-215	Legionella pneumophila	Legionella pneumophila
T01-216	Candida albicans<br>Candida tropicalis<br>Candida glabrata<br>Acinetobacter baumannii	Candida albicans<br>Candida tropicalis<br>Candida glabrata<br>Acinetobacter baumannii
T01-217	Candida albicans<br>Staphylococcus aureus<br>Acinetobacter baumannii	Candida albicans<br>Staphylococcus aureus<br>Acinetobacter baumannii
T01-218	Acinetobacter baumannii<br>Staphylococcus aureus	Acinetobacter baumannii
T01-219	Haemophilus influenzae	Haemophilus influenzae
T01-220	Candida tropicalis	Candida tropicalis
T01-221	Klebsiella pneumoniae	Klebsiella pneumoniae
T01-222	Pneumocystis jirovecii<br>Acinetobacter baumannii	Pneumocystis jirovecii<br>Acinetobacter baumannii
T01-223	Klebsiella pneumoniae	Klebsiella pneumoniae
T01-224	Pneumocystis jirovecii<br>Candida albicans	Pneumocystis jirovecii<br>Candida albicans
T01-225	Aspergillus fumigatus<br>Pseudomonas aeruginosa<br>Staphylococcus aureus	Staphylococcus aureus<br>Aspergillus fumigatus<br>Pseudomonas aeruginosa
T01-226	Pneumocystis jirovecii<br>Candida albicans<br>Pseudomonas aeruginosa	Pneumocystis jirovecii<br>Candida albicans<br>Pseudomonas aeruginosa

T01-227	Aspergillus fumigatus<br>Acinetobacter baumannii	Aspergillus fumigatus<br>Acinetobacter baumannii
T01-228	Acinetobacter baumannii	Acinetobacter baumannii
T01-229	Aspergillus fumigatus<br>Acinetobacter baumannii	Aspergillus fumigatus<br>Acinetobacter baumannii
T01-230	Candida glabrata	Candida glabrata
T01-231	Acinetobacter baumannii<br>Pseudomonas aeruginosa	Acinetobacter baumannii<br>Pseudomonas aeruginosa
T01-232	Pseudomonas aeruginosa	Pseudomonas aeruginosa
T01-233	Klebsiella pneumoniae<br>Candida albicans<br>Staphylococcus aureus<br>Candida glabrata<br>Acinetobacter baumannii	Klebsiella pneumoniae<br>Candida albicans<br>Staphylococcus aureus<br>Candida glabrata<br>Acinetobacter baumannii
T01-234	Haemophilus influenzae<br>Moraxella catarrhalis	Haemophilus influenzae<br>Moraxella catarrhalis
T01-235	Haemophilus influenzae	Haemophilus influenzae
T01-236	Candida albicans<br>Pseudomonas aeruginosa	Candida albicans<br>Pseudomonas aeruginosa

T01-237	Klebsiella pneumoniae<br>Candida albicans<br>Acinetobacter baumannii	Klebsiella pneumoniae<br>Candida albicans<br>Acinetobacter baumannii
T01-238	Candida albicans<br>Klebsiella pneumoniae<br>Staphylococcus aureus	Klebsiella pneumoniae<br>Candida albicans<br>Staphylococcus aureus
T01-239	Staphylococcus aureus	Staphylococcus aureus
T01-240	Pneumocystis jirovecii<br>Acinetobacter baumannii	Pneumocystis jirovecii<br>Acinetobacter baumannii
T01-241	Candida albicans<br>Acinetobacter baumannii	Candida albicans<br>Acinetobacter baumannii
T01-242	Pneumocystis jirovecii	Pneumocystis jirovecii
T01-243	Pseudomonas aeruginosa	Negative
T01-244	Pseudomonas aeruginosa	Pseudomonas aeruginosa
T01-245	Acinetobacter baumannii	Acinetobacter baumannii
T01-246	Candida albicans	Candida albicans
T01-247	Klebsiella pneumoniae<br>Acinetobacter baumannii<br>Pseudomonas aeruginosa	Acinetobacter baumannii<br>Pseudomonas aeruginosa
T01-248	Acinetobacter baumannii	Acinetobacter baumannii
T01-249	Acinetobacter baumannii	Acinetobacter baumannii
T01-250	Candida albicans	Candida albicans

T01-251	Klebsiella pneumoniae	Klebsiella pneumoniae
T01-252	Candida albicans<br>Candida tropicalis<br>Pseudomonas aeruginosa<br>Staphylococcus aureus	Candida albicans<br>Staphylococcus aureus<br>Candida tropicalis<br>Pseudomonas aeruginosa
T01-253	Candida albicans<br>Pseudomonas aeruginosa	Candida albicans
T01-254	Streptococcus pneumoniae<br>Pseudomonas aeruginosa	Streptococcus pneumoniae<br>Pseudomonas aeruginosa
T01-255	Candida albicans<br>Candida tropicalis	Candida albicans<br>Candida tropicalis
T01-256	Candida albicans<br>Pseudomonas aeruginosa	Candida albicans<br>Pseudomonas aeruginosa
T01-257	Pseudomonas aeruginosa<br>Staphylococcus aureus	Staphylococcus aureus<br>Pseudomonas aeruginosa
T01-258	Streptococcus pneumoniae<br>Moraxella catarrhalis<br>Staphylococcus aureus	Staphylococcus aureus<br>Streptococcus pneumoniae<br>Moraxella catarrhalis
T01-259	Candida albicans<br>Acinetobacter baumannii	Candida albicans<br>Acinetobacter baumannii
T01-260	Klebsiella pneumoniae	Klebsiella pneumoniae
T01-261	Candida glabrata<br>Acinetobacter baumannii	Candida glabrata<br>Acinetobacter baumannii

T01-262	Staphylococcus aureus	Staphylococcus aureus
T01-263	Acinetobacter baumannii	Acinetobacter baumannii
T01-264	Staphylococcus aureus	Staphylococcus aureus
T01-265	Haemophilus influenzae	Haemophilus influenzae
T01-266	Aspergillus fumigatus<br>Pneumocystis jirovecii<br>Staphylococcus aureus	Pneumocystis jirovecii<br>Staphylococcus aureus<br>Aspergillus fumigatus
T01-267	Klebsiella pneumoniae	Klebsiella pneumoniae
T01-268	Candida albicans	Candida albicans
T01-269	Pseudomonas aeruginosa	Pseudomonas aeruginosa
T01-270	Cryptococcus neoformans<br>Candida albicans<br>Acinetobacter baumannii	Candida albicans<br>Acinetobacter baumannii<br>Cryptococcus neoformans
T01-271	Pseudomonas aeruginosa	Pseudomonas aeruginosa
T01-272	Candida tropicalis	Candida tropicalis
T01-273	Klebsiella pneumoniae	Klebsiella pneumoniae
T01-274	Candida albicans	Candida albicans
T01-275	Candida albicans	Candida albicans

T01-276	Candida albicans<br>Acinetobacter baumannii<br>Pseudomonas aeruginosa	Candida albicans<br>Acinetobacter baumannii<br>Pseudomonas aeruginosa
T01-277	Aspergillus fumigatus	Aspergillus fumigatus
T01-278	Candida albicans<br>Staphylococcus aureus	Candida albicans<br>Staphylococcus aureus
T01-279	Klebsiella pneumoniae	Klebsiella pneumoniae
T01-280	Klebsiella pneumoniae	Klebsiella pneumoniae
T01-281	Klebsiella pneumoniae	Klebsiella pneumoniae
T01-282	Pneumocystis jirovecii	Pneumocystis jirovecii<br>Acinetobacter baumannii
T01-283	Pneumocystis jirovecii<br>Candida albicans<br>Candida tropicalis<br>Candida glabrata	Pneumocystis jirovecii<br>Candida albicans<br>Candida tropicalis<br>Candida glabrata
T01-284	Candida albicans<br>Klebsiella pneumoniae	Klebsiella pneumoniae<br>Candida albicans
T01-285	Staphylococcus aureus	Staphylococcus aureus
T01-286	Candida albicans<br>Acinetobacter baumannii	Candida albicans<br>Acinetobacter baumannii
T01-287	Candida albicans	Candida albicans
T01-288	Klebsiella pneumoniae	Klebsiella pneumoniae

T01-289	Candida albicans<br>Pseudomonas aeruginosa	Candida albicans<br>Pseudomonas aeruginosa
T01-290	Negative	Candida albicans
T01-291	Negative	Negative
T01-292	Candida tropicalis	Candida tropicalis
T01-293	Pneumocystis jirovecii<br>Candida albicans	Pneumocystis jirovecii<br>Candida albicans
T01-294	Pneumocystis jirovecii<br>Pseudomonas aeruginosa	Pneumocystis jirovecii<br>Pseudomonas aeruginosa
T01-295	Aspergillus fumigatus<br>Pneumocystis jirovecii<br>Candida tropicalis<br>Acinetobacter baumannii<br>Staphylococcus aureus	Pneumocystis jirovecii<br>Staphylococcus aureus<br>Aspergillus fumigatus
T01-296	Streptococcus pneumoniae<br>Pseudomonas aeruginosa	Streptococcus pneumoniae<br>Pseudomonas aeruginosa
T01-297	Negative	Negative
T01-298	Haemophilus influenzae	Haemophilus influenzae
T01-299	Negative	Negative
T01-300	Negative	Negative
T01-301	Pseudomonas aeruginosa	Pseudomonas aeruginosa
T01-302	Negative	Negative
T01-303	Negative	Negative
T01-304	Acinetobacter baumannii<br>Pseudomonas aeruginosa	Acinetobacter baumannii<br>Pseudomonas aeruginosa
T01-305	Negative	Negative
T01-306	Negative	Negative
T01-307	Negative	Negative

T01-308	Negative	Negative
T01-309	Pseudomonas aeruginosa	Pseudomonas aeruginosa
T01-310	Klebsiella pneumoniae	Klebsiella pneumoniae
T01-311	Haemophilus influenzae	Haemophilus influenzae
T01-312	Candida albicans	Candida albicans
T01-313	Negative	Negative
T01-314	Negative	Negative
T01-315	Candida albicans<br>Acinetobacter baumannii<br>Pseudomonas aeruginosa	Candida albicans<br>Acinetobacter baumannii<br>Pseudomonas aeruginosa
T01-316	Staphylococcus aureus	Staphylococcus aureus
T01-317	Pseudomonas aeruginosa	Pseudomonas aeruginosa
T01-318	Aspergillus fumigatus<br>Klebsiella pneumoniae	Klebsiella pneumoniae
T01-319	Aspergillus fumigatus<br>Pneumocystis jirovecii	Pneumocystis jirovecii<br>Aspergillus fumigatus
T01-320	Acinetobacter baumannii	Klebsiella pneumoniae<br>Acinetobacter baumannii
T01-321	Klebsiella pneumoniae	Klebsiella pneumoniae
T01-322	Klebsiella pneumoniae<br>Pseudomonas aeruginosa	Klebsiella pneumoniae<br>Pseudomonas aeruginosa
